# Fecal carriage of extended spectrum β-lactamase producing *Escherichia coli* and *Klebsiella pneumoniae* after urinary tract infection – A three year prospective cohort study

**DOI:** 10.1371/journal.pone.0173510

**Published:** 2017-03-07

**Authors:** Silje B. Jørgensen, Arne Søraas, Arnfinn Sundsfjord, Knut Liestøl, Truls M. Leegaard, Pål A. Jenum

**Affiliations:** 1 Department of Laboratory Medicine, Section for Medical Microbiology, Vestre Viken Hospital Trust, Bærum, Norway; 2 Department of Clinical Microbiology and Infection Control, Akershus University Hospital, Lørenskog, Norway; 3 Department of Infectious Diseases, Oslo University Hospital, Oslo, Norway; 4 Norwegian National Advisory Unit on Detection of Antimicrobial Resistance, Department of Microbiology and Infection Control, University Hospital of North Norway, Tromsø, Norway; 5 Research Group for Host-Microbe Interactions, Department of Medical Biology, Faculty of Health Sciences, University of Tromsø, Tromsø, Norway; 6 Department of Informatics, University of Oslo, Oslo, Norway; 7 Institute of Clinical Medicine, University of Oslo, Oslo, Norway; Universite Paris Descartes, FRANCE

## Abstract

We have performed a prospective cohort study to investigate the duration of and risk factors for prolonged fecal carriage of ESBL-producing *Escherichia coli* or *Klebsiella pneumoniae* in patients with community acquired urinary tract infection caused by these bacteria. From 2009 to 2011, 101 Norwegian patients were recruited. Stool swabs and questionnaires were collected every three months for one year and at the end of the study in 2012. Information on antibiotic prescriptions was collected from the Norwegian Prescription Database. Stool samples were cultured directly on ChromID ESBL agar as well as in an enrichment broth, and culture positive isolates were examined by *bla*_CTX-M_ multiplex PCR. Isolates without *bla*_CTX-M_ were investigated for alternative ESBL-determinants with a commercial microarray system. Time to fecal clearance of ESBL producing *Enterobacteriaceae* was also analysed using Kaplan-Meier estimates. Uni- and multivariate logistic regression was used to compare groups according to previously described risk factors. The ESBL point prevalence of fecal carriage were 61% at 4 months, 56% at 7 months, 48% at 10 months, 39% at 13 months, 19% after two years, and 15% after three years or more. We found no correlation between duration of carriage, comorbidity, antibiotic use or travel to ESBL high-prevalence countries. Prolonged carriage was associated with *E*. *coli* isolates of phylogroup B2 or D. Importantly, comparative MLST and MLVA analyses of individual paired urine and fecal *E*. *coli* isolates revealed that ESBL production commonly occurred in diverse strains within the same host. When investigating cross-transmission of ESBL producing bacteria in health care institutions, this notion should be taken into account.

## Introduction

Extended spectrum β-lactamase producing *Enterobacteriaceae* (ESBL-E) are an important cause of nosocomial and community acquired infections [1,2]. The spread of ESBL-E is facilitated by their reservoir in the gut of healthy humans and animals, and in water [3]. Certain clones, such as the epidemic *Escherichia coli* strain CTX-M 15 ST131, have successfully disseminated internationally [4]. The increasing threat of antibiotic resistance has prompted surveillance of drug-resistant bacteria from stool samples as a tool to track their migration and to implement infection control measures [5–7].

Several risk factors for the acquisition of intestinal ESBL-E have been described, and they seem to vary between low- and high-prevalence countries [8–14]. However, there are few studies regarding the time course of carriage of ESBL-E in the intestine and the factors that may influence duration of carriage [12,15–18]. Knowledge about carriage duration is important for implementation of adequate infection control measures, and essential in the construction of mathematical models in our understanding of the epidemiology of ESBL-E dissemination.

Previous studies on clinical patients conclude that fecal carriage seems to be common several months after an infection with ESBL-E, and that ESBL-E negative stool samples occur intermittently during colonisation. However, they identify different risk factors for prolonged carriage, such as urinary catheter use [16], immobility [17], infection with *E*. *coli* strains belonging to phylogroup B2 (which includes ST131) [15,18] and CTX-M group 1 enzymes [15].

We have previously shown that travel to high-prevalence countries, antibiotic use, diabetes and fresh-water swimming are independent risk factors for UTI caused by ESBL-E [8]. In this prospective cohort study of the same patients, we examined the duration of fecal carriage of ESBL-E, potential risk factors for prolonged carriage and the clonal relatedness between paired ESBL-producing *E*. *coli* urinary and fecal strains.

### Ethics statement

The study was approved by the Regional Committee for Medical and Health Research Ethics December 11^th^ 2008 (reference number: 2009/2037 BS-08901b). It is registered in the ClinicalTrials.gov registration system, registration ID NCT01838213 in 2013. As this is an epidemiological observational study, registration as a clinical trial was not considered as necessary at the beginning of the project in 2009, but was done in April 2013. The authors confirm that all trials on going and related to this project are registered. The included population has also been part of two previously published case control studies by Søraas et al on risk factors for urinary tract infections caused by ESBL-E [8], and on the effects of pivmecillinam treatment of these infections [19], available as supplementary files S7 and S8. The complete and detailed study protocol as approved by the ethics committee before the study began is available as supporting information S5 and S6. An addition to the protocol was submitted and approved by the ethics committee May 8 2012 [S1 Text, S2 Text]. This addition excluded, the study followed the original protocol.

## Materials and methods

### Study setting

Vestre Viken Hospital Trust is situated in a mixed urban and suburban region in the South-Eastern part of Norway, and has a catchment area of 475.000 inhabitants. Our Department of Medical Microbiology analyses samples from both in- and out-patients. Inclusion of patients started in March 2009, and the last samples were collected in August 2012.

### Data collection

This prospective cohort study included patients above 18 years, with a urine culture yielding >10,000 CFU/ml ESBL producing *E*. *coli* or *K*. *pneumoniae*. Exclusion criteria were as follows: 1) residency in Norway for less than one year, 2) healthcare associated urinary tract infection, 3) inability to answer questionnaires, and 4) previous ESBL-E infection. All participants gave a written consent of participation.

**Fig 1:**
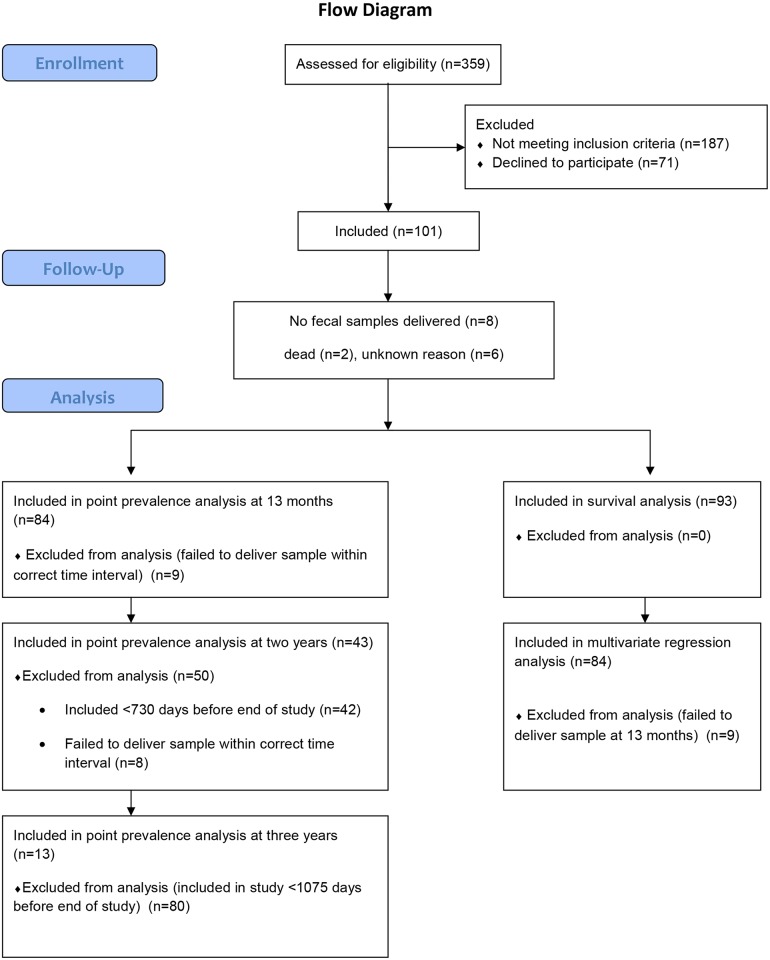
Flow diagram of study patient inclusion.

Patients were recruited between March 10^th^ 2009 and March 21^st^ 2011. At inclusion, the patients were asked to deliver a fecal sample and undergo a structured interview regarding possible risk factors for ESBL positive UTI [8]. They were then followed prospectively with new fecal samples and questionnaires every 3 months for one year. In May 2012, all participants were asked to deliver a new stool sample and questionnaire, regardless of their time since inclusion (Fig 1). The questionnaires concerned occurrence of new urinary tract infections, international travel or residency, antibiotic use, and foreign visitors in the household. To secure optimal sampling, the participants were given instructions to obtain the fecal sample from used toilet paper by use of cotton swabs transported on Amies medium and ensure that visible fecal material was deposited on the swab.

Purchasing dates, types and amount of antibiotic drugs sold to the patients during the study period were obtained from The Norwegian Prescription Database, and antibiotic use during hospitalization was recorded from the patients’ medical records. Only systemic drugs with ATC-codes in group J were included, including antimycotics but excluding methenamine, antiviral drugs and vaccines. As a measurement for antibiotic consumption, defined daily doses (DDDs) for each drug is a useful tool [20]. The measurement unit DDD is defined as the assumed average maintenance dose per day for a drug used for its main indication in adults.

### Laboratory methods

#### Identification and ESBL-detection in urine isolates

Urine cultivation, bacterial identification and antibiotic susceptibility testing were performed using ChromID ESBL agar and the VITEK-2 system (both from bioMérieux, Marcy l’Etoile, France). For antimicrobial susceptibility interpretations we used EUCAST breakpoints (http://www.eucast.org), and for ESBL/AmpC-confirmation the E-test system (AB-Biodisk, BioMérieux). ESBL genotype analysis was performed using PCR for *bla*_CTX-M_ detection and group assignment as described [21]. Urine isolates negative for *bla*_CTX-M_ were analysed using conventional *bla*_TEM_ and *bla*_SHV_ PCR and sequencing [22].

#### Identification and ESBL-detection in fecal isolates

The fecal samples were cultured directly on lactose (growth control) and selective chrome agar, and also in selective brain heart infusion enrichment broth containing cefotaxime 2.5 g/L, with sub cultivation on chrome agar. Isolates with a colour indicating *E*. *coli* or *Klebsiella sp*. were included for further analysis. Oxidase negative isolates with atypical colours were identified by VITEK-2. All fecal *E*. *coli* and *Klebsiella* isolates with an ESBL or AmpC phenotype were investigated for *bla*_CTX-M_ with the same method as the urine isolates.

Species identification of the *bla*_CTX-M_ negative isolates was confirmed by MALDI-TOF (Bruker Daltonics, Germany). Confirmed ESBL or AmpC phenotypes were analysed by microarray Check-MDR CT101 (Check-Points B.V., Wageningen, the Netherlands) targeting *bla*_TEM_, *bla*_SHV,_ *bla*_NDM_, *bla*_KPC_, as well as the following plasmid mediated AmpC variants: CMY, DHA, FOX, ACC, ACT, MIR and MOX.

#### MLVA, MLST-typing and phylogroup assignment of *E*. *coli* isolates

Clonal relatedness between fecal and urinary ESBL-producing *E*. *coli* was examined by a multilocus variable number of tandem repeats assay (MLVA) protocol originally described by Lindstedt et al and modified by Løbersli et al [23,24]. Briefly, PCR-products were subjected to capillary electrophoresis, and each peak was identified according to colour and size by GeneMapper software (Applied Biosystems, Foster City, CA, USA). Allele numbers were assigned according to fragment sizes as previously described [24]. Character values were entered into BioNumerics (Applied Maths, Saint-Martens-Letem, Belgium), and dendrograms were constructed using categorical coefficients and the Ward algorithm. A standard minimum spanning tree was generated using categorical coefficients together with the single and double locus variance priority rules. Whole genome sequencing by Illumina technology (Illumina, San-Diego, USA) generating 150 base pairs paired-end reads was performed on all *E*. *coli* urine isolates. Identification of eight housekeeping genes with corresponding alleles and sequence types (STs) were determined by using the database at Center for Genomic Epidemiology [25,26]. To establish whether the urine isolates belonged to phylogroup B2 or D, we conducted BLAST analysis (www.blast.ncbi.nlm.nih.gov) seeking alignment with *chuA* [27].

### Statistical analysis and risk factor definitions

Sample size was calculated to fit the previously published case-control study [8]. Point prevalence rates were calculated for each three-month period. Carriage survival was estimated in a Kaplan-Meier curve. As many patients with negative samples later became positive in new samples, we found it appropriate to define ESBL-E clearance as two consecutive negative samples, and to present an estimate where the event end-point is set to occur at the first negative sample if the following sample also is negative. For further risk factor analysis, we used carriage at 13 months after inclusion as the dependent variable in uni- and multivariable analyses. Univariate analyses were performed using Student’s *t* test or Pearson’s chi-square test when appropriate. Multivariate analyses were preceded by estimation of correlation between risk factors in a multiple logistic regression model. To reveal significant risk factors for persisting ESBL-E carriage at 13 months, we conducted backwards elimination of variables, starting by including previously described risk factors related to microbe- and patient characteristics. There was only one variable significantly associated with prolonged carriage. Thus, an exploratory model of hypothetical risk factors is presented [28]. The association between potential risk factors and carriage duration was quantified by odds ratio (OR) with 95% confidence interval (CI). A P-value < = 0.05 was considered significant. The continuous variables have been dichotomized, but the main results were robust to alternative operationalizations. Statistical analyses were conducted using PASW statistics software, version 21.0 (IBM SPSS, IL, USA) and Stata Statistical Software release 13 (StataCorp, TX, USA).

## Results

Approximately 28,000 urine samples from 15,000 unique patients were submitted to our department during the inclusion period. A total of 359 (1.3%) samples yielded ESBL positive *E*. *coli* or *K*. *pneumoniae*. After exclusion, 172 subjects with ESBL-E UTI were invited to participate, and 101 cases (56%; *E*. *coli* (n = 96) and *K*. *pneumoniae* (n = 5)) were included; women (n = 89) and men (n = 12), with a median age of 55 years (range 18–95). The mean age was 2.8 years higher for participants than for non-participants, otherwise no statistically significant difference was found between groups [8]. Two patients died during the study, and six others were lost to follow-up. All samples from these patients were excluded in the statistical analyses. Within the first 13 months of inclusion, eleven patients report to have received antibiotic treatment in hospital. In all cases, this treatment took place within the first three months after inclusion. We asked for 595 fecal samples in total, and received 519 (87%). Of these, 247 samples (48%) contained one or more morphologically distinct colonies of ESBL-E. Molecular detection of *bla* was performed on 267 morphologically distinct fecal isolates from 77 patients of which 237 (89%) were *E*. *coli*. The enrichment broth yielded only two additional positive samples compared with direct culture, of which one was an AmpC-phenotype *E*. *coli*. The follow-up time varied from 12 to 41 months, with an average of 27 months. Ninety-three patients were followed for one year. The follow-up time was dictated by the time of inclusion. Hence, 44 patients were eligible for two years follow up, from which we received final samples from 43 (98%). 13 patients were eligible for three years follow up, and we received samples from all of these. A comparison of the two-year and three-year groups to the total cohort is presented in Table 1.

**Table 1:** Comparison of participants according to follow-up time.

	Total cohort, one year follow-up, (n = 93)	Two years follow-up, (n = 44)	Three years follow-up, (n = 13)
Age	Mean 55Median 55 Range 18–95	Mean 55Median 56Range 19–92	Mean 56Median 56Range 39–92
Male sex	12 (13%)	6 (10%)	3 (23%)
Charlson comorbidity index score [29]	Mean 0,92Median 0Range 0–10	Mean 0,74Median 0Range 0–10	Mean 0,42Median 0Range 0–1
Chronic UTI	20 (21.5%)	9 (20.5%)	3 (23.1%)
Antibiotics*	66 (71%)	31 (70.5%)	8 (61.5%)
Travel to Asia, Africa or Middle-East within first 15 months after UTI	20 (21.5%)	10 (22.7%)	5 (38.5%)
Number of household members	Mean 2.33Median 2Range 1–8	Mean 2.39Median 2Range 1–8	Mean 2.46Median 2Range 1–5
*E*. *coli* phylogroup B2/D in urine	65 (70%)	33 (75%)	10 (77%)

* = Any antibiotic received between 14 days and 13 months after positive urinary sample.

### ESBL genotypes

In the 101 urine isolates *bla*_CTX-M_ group 1, group 9 and ESBL-type *bla*_SHV_ were present in 66 (65%), 30 (30%) and in five (5%) of the urinary isolates respectively. The ESBL-genotype proportions were quite similar in fecal isolates; 61%, 35% and 4%. We also detected one *bla*_KPC_-positive, one *bla*_DHA_ and one *bla*_CMY-II_−/*bla*SHV−positive strain.

### Travel

Of the 93 patients who completed the study, 73% (n = 68) travelled outside of the Nordic countries during the study period. The most frequent destinations were Spain (n = 46), Africa and/or Asia (n = 17), and America (n = 6). One patient was permanently living in India. The participants had between zero and 343 travel days (mean 14 days).

### Antibiotic use

Excluding the first two weeks after the UTI diagnosis, a total of 74 patients (80%) received antibiotics during the study period. The most commonly used drug was pivmecillinam followed by nitrofurantoin. There were 16 reported cases of antibiotic treatment in hospitals. According to the Norwegian Prescription Database, a total number of 3,973 defined daily doses (DDDs) of antibiotics were purchased by the patients throughout the study, with a range of zero to 732 (mean 54, median 23) DDDs per patient for a total number of patient days of 76,265. This equals 52 DDDs/1,000 patient days. The majority of the consumed antibiotics were those commonly used for the treatment of UTI (data in S1 Fig. Antibiotic consumption). On-going antibiotic therapy, or antibiotic use within 5, 10, 30 or 90 days prior to sampling did not correlate with the sample being positive for ESBL-E.

### Duration of carriage

**Fig 2:**
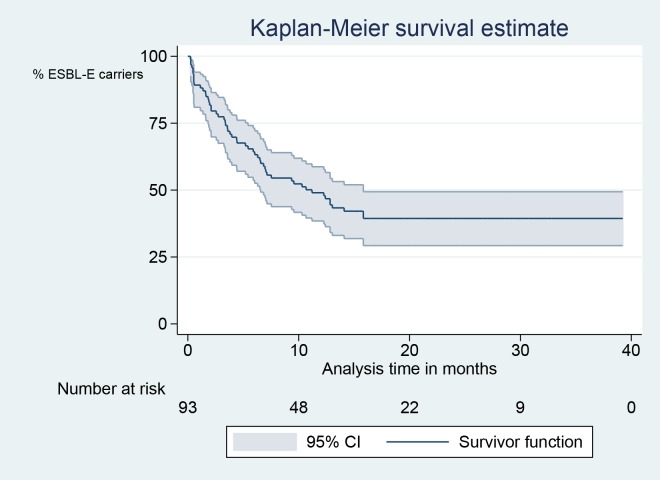
Kaplan-Meier survival plot. Fecal ESBL-E clearance is defined to occur at the first negative sample if the following sample also is negative.

The observed prevalence carriage rate was 53/87 (61%) at 4 months, 48/86 (56%) at 7 months, 40/84 (48%) at 10 months, 33/84 (39%) at 13 months, 8/43 (19%) after two years or more, and 2/13 (15%) after three years or more; 13 of 93 patients (14%) with negative samples became positive again in later samples. In Fig 2, the ESBL-E clearance rate is illustrated in a Kaplan-Meier survival curve (Fig 2). In this analysis, 50% of the participants are negative at 12 months. Only three patients (3%) became positive after two or more consecutive negative samples. In these cases, the last isolates were a new ESBL-producing species (n = 1), or had a different *E*. *coli* MLVA genotype (n = 2). All three had travelled to high-endemic countries after the second negative sample but before the new positive sample.

Phylogroup, as defined by presence of *chuA* was the only variable significantly associated with prolonged carriage. An exploratory model of hypothetical risk factors is presented (Table 2).

**Table 2:** Multiple logistic regression model of potential risk factors for ESBL-E carriage 13 months after urinary tract infection.

			Univariate analysis			Multivariate analysis		
Variable	ESBL-positive (n = 44)	ESBL-negative (n = 39)	OR	95% CI	P	OR	95% CI	P
**Age > 65 years**	18	13	1.4	0.59–3.5	0.426	1.8	0.60–5.4	0.294
**Male sex**	5	5	0.9	0.24–3.4	0.872	0.85	0.18–3.9	0.833
**Chronic urinary tract infection**	11	9	1.2	0.42–3.2	0.788	1.0	0.32–3.3	0.957
**Charlson score**[29] **>2**	7	4	1.7	0.46–6.3	0.426	1.7	0.39–7.2	0.480
**Number of household members >3**	8	5	1.6	0.46–5.2	0.474	1.5	0.37–5.8	0.594
***E*. *coli* phylogroup B2 or D in urine**	37	24	3.3	1.2–9.3	0.023	3.8	1.2–12.6	0.027
**CTX-M group 1 in urine isolate**	26	28	0.62	0.25–1.5	0.299	0.70	0.26–1.9	0.492
Antibiotics*	32	31	0.77	0.27–2.1	0.614	0.97	0.32–3.0	0.958
**Travel to Asia, Africa or Middle-East**	10	10	0.92	0.32–2.4	0.807	0.778	0.25–2.4	0.661

Clearance is defined as two consecutive negative samples. OR = odds ratio for ESBL persistence, CI = confidence interval.

* Any antibiotic received between 14 days and 13 months after positive urinary sample.

#### MLVA-analysis of selected ESBL-producing urinary and fecal *E*. *coli* isolates

**Fig 3:**
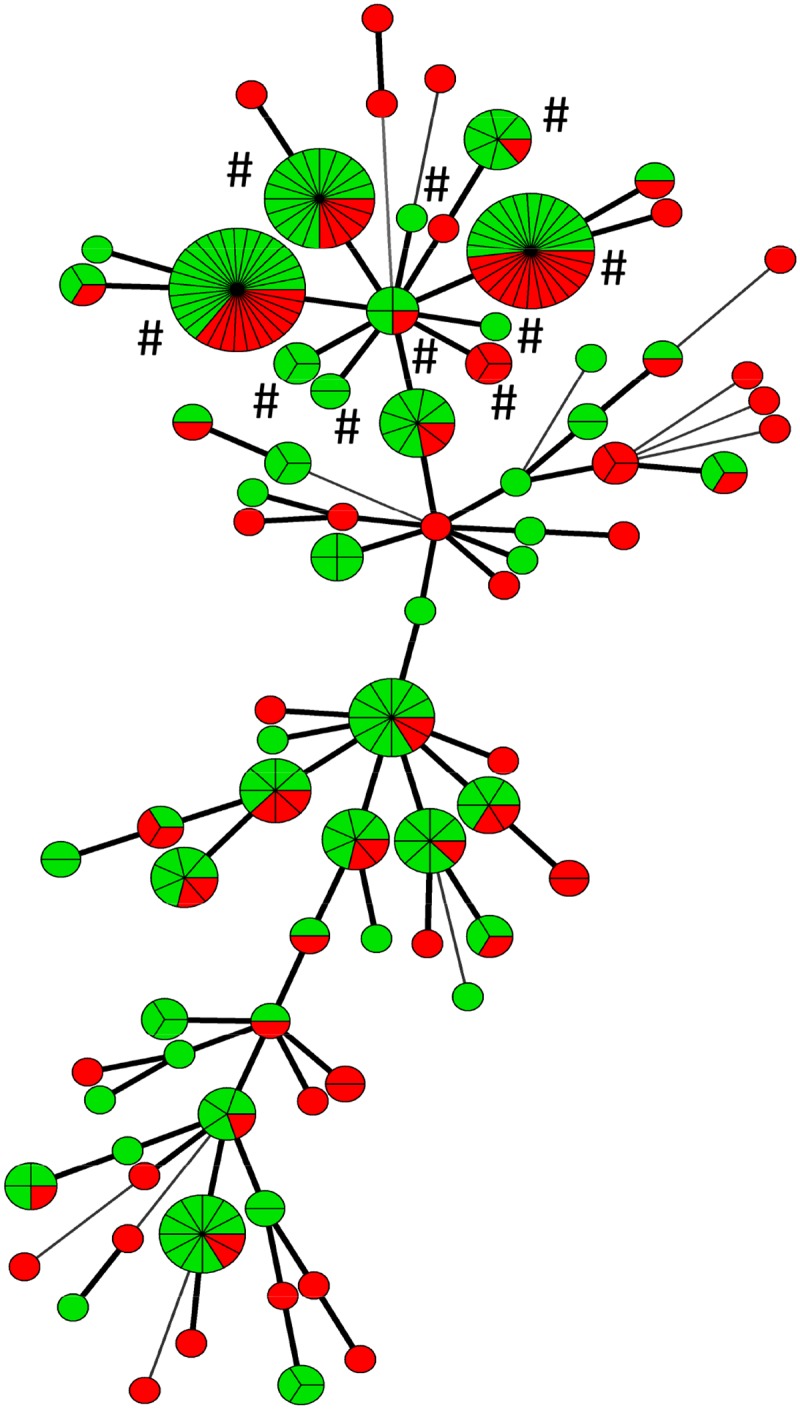
Minimum spanning tree of *E*. *coli* urine isolates (97 isolates from 95 patients) and fecal isolates (168 isolates from 35 patients). Urine isolates are marked with red colour, fecal isolates with green colour. Short, fat lines between two nodes signify that the strains in each node have a different band number in only one locus. Long, thin lines between two nodes signify different band numbers in more than one locus. ST131 strains are marked with #.

All strains from patients who were ESBL-E carriers for more than 13 months (n = 35), were selected for MLVA. These long-term carriers delivered 168 *E*. *coli* fecal isolates in total (range 1–7, median 5). Among the *E*. *coli* urine isolates (n = 97) of all participants and fecal isolates (n = 168) from the 35 long-term carriers, we identified 80 distinct MLVA-types; three large clusters containing 20, 27 and 31 isolates respectively, 23 clusters containing between three and 12 isolates, 10 clusters with two isolates, and 44 singletons (Fig 3). Both fecal and urine isolates are present in 24 of the clusters. The three largest clusters and twelve of the isolates closely related to them, shared a MLVA profile that corresponds to ST131 (S1 Data) [30].

**Fig 4:**
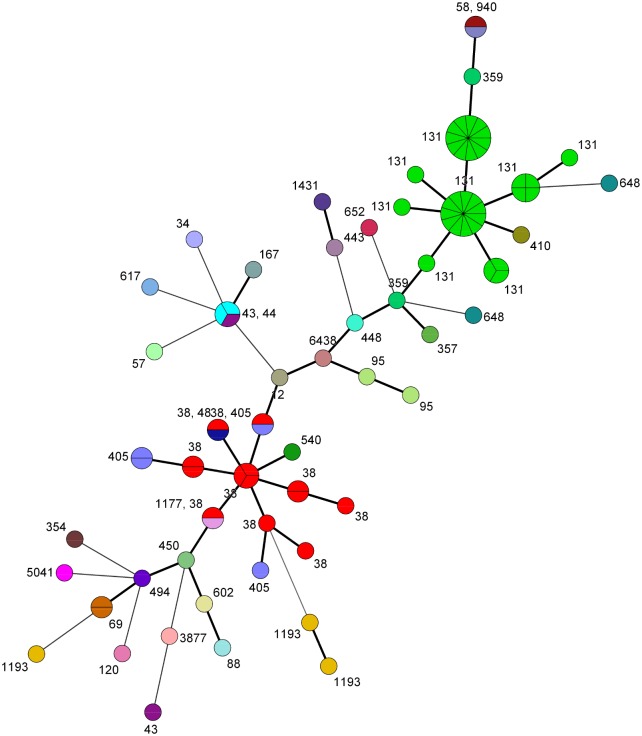
Minimum spanning tree of *E*. *coli* urine isolates (97 isolates from 95 patients) represented as circles with STs labelled and represented with different colours. Short, fat lines between two nodes signify that the strains in each node have a different band number in only one locus. Long, thin lines between two nodes signify different band numbers in more than one locus. Each ST is represented with a distinct colour.

We identified 35 different STs and 51 different MLVA profiles in the urine isolates. The largest group contained 27 urine strains of ST131. The second largest group contained 13 isolates that belonged to ST38. The other STs were present in four strains or less. Correlation of MLVA profiles and STs is illustrated in Fig 4 and listed in S1 Data.

**Fig 5:**
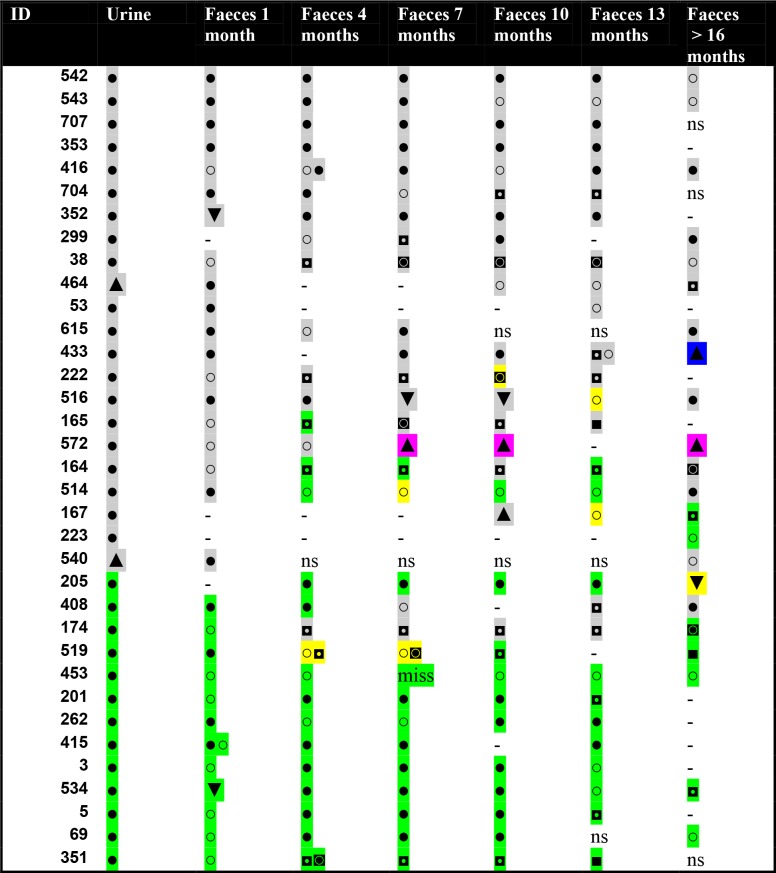
Species, *E*. *coli* MLVA types and ß-lactamase enzymes in long-term fecal carriers (n = 35). Each ß-lactamase enzyme is marked with a distinct colour: grey square = CTX-M group 1, green square = CTX-M group 9, pink square. = SHV, blue square. = KPC, yellow square = several different ß-lactamases. Variation in *E*. *coli* MLVA-type is marked by separate symbols: ● = *E*. *coli* first MLVA-type, ○ = *E*. *coli* second MLVA-type, ◘ = *E*. *coli* third MLVA-type, ◙ = *E*. *coli* fourth MLVA-type, ■ = *E*. *coli* fifth MLVA-type. Species other than *E*. *coli* are marked by triangles: ▲ = *K*. *pneumoniae*, ▼ = *Enterobacteriaceae* other than *E*. *coli* or *K*. *pneumonia*. ns = no sample, - = no ESBL-producing isolate found, miss = lost sample.

Of the 35 long-term carriers, only two participants (6%) had the same MLVA-type in all samples. Two different types were present in 15 patients (43%), while there were three, four and five types in 12 (34%), four (11%) and three (9%) patients respectively. Ninety-eight of 168 positive fecal samples (58%) contained either a different ESBL producing species (5%, n = 8) or an *E*. *coli* with a different MLVA-type than the urinary isolate (54%, n = 90). Fifteen patients (45%) had the same *E*.*coli* MLVA-type in urine as in the first fecal sample. Changes in ß-lactamases within the same MLVA-type in the same patient occurred in four cases (11%). Changes in species, β-lactamases and MLVA-types in each patient are illustrated in Fig 5.

## Discussion

We have examined fecal ESBL-E carriage duration in patients with UTI caused by ESBL-producing *E*. *coli* or *K*. *pneumoniae*. Ninety-three patients were followed for one year, 44 patients for two years, and 13 patients for three years or more. We recorded known risk factors for ESBL-carriage and characterized the ESBL-producing *E*. *coli* isolates by MLVA.

Patient compliance in follow-up was successful, as 87% of requested fecal samples were delivered. We found that 44% were still ESBL-E carriers after one year by using survival analysis defining clearance as two consecutive negative samples. This may be an overestimation of ESBL-E clearance time, as the actual elimination of the ESBL-E carriage is unlikely to fall upon the exact date of the first negative sample. One could argue that the end-point should be set between the last positive sample and the first negative sample. On the other hand, one could also argue that the end-point should be set at the second negative sample, because a second sample is needed to confirm negativity in a clinical setting. The ESBL-E elimination rate estimate curve flattens in the two last years of the study, because we have set the end-point to occur at the first of two consecutive negative samples. Consequently, the latest possible event occurs at 13 months. When interpreting the curve, it is also important to remember that the interval between the 13-months sample and the last sample varies considerably between the participants. Simple point prevalence analysis with clearance defined as only one negative sample, reveals slightly lower positivity rates, as some patients delivered transient negative samples. Consequently, we recommend that at least two negative samples are obtained to verify ESBL-E clearance. To investigate the ideal time interval between these samples was beyond the scope of this study.

Our clearance rates are in accordance with previous studies from Sweden, Slovenia and France [15,17,31,32]. Lack of detection of ESBL-E does not confirm absence of ESBL-E, but the risk of further spread can probably be regarded as low. Self-sampling may influence the sensitivity of microbiological tests, but in this study bacterial growth was obtained from all but one sample, in which case a repeated sample was obtained. Contrary to other studies [33], culture in selective enrichment broth did not give a significantly higher yield of ESBL-E. This could be due to differences in media composition, lack of vancomycin to inhibit growth of *Enterococci*, and the higher concentration of cefotaxime in our broth.

Apart from the long duration of ESBL-E carriage, our most important finding is the high genetic diversity among ESBL-producing *E*. *coli* strains within the same individual. Changes in MLVA-type occurred during long term fecal carriage in 89% of the patients. A patient’s fecal MLVA-type differed from the urinary type in 54% of the samples. There is less concordance between urine strains and fecal strains than shown in previous studies of patients with non-ESBL *E*. *coli* UTI [34], but the differences may be due to different typing methods. The seven loci MLVA protocol which we have used, gives a differentiation similar to pulsed-field gel electrophoresis [23,35]. In the MLVA minimum spanning tree, we observe a nice clustering of the dominating STs (ST131 and ST38), as previously shown by others [30].

The ESBL encoding elements are most often situated on plasmids that are horizontally transmitted between bacteria. Thus, it is likely that spread to new strains and species within the same patient increases during prolonged carriage. Comparative studies of ESBL-E within households and in long term care institutions also show genetic diversity in carriage strains [14,35,36]. For the majority of our patients, the CTX-M group is the same even if the MLVA-type changes. This observation supports the notion of horizontal gene transfer rather than the acquisition of a new ESBL-producing strain. We performed MLVA-analysis of morphologically distinct colonies from each positive fecal sample, but only on one isolate of each morphotype. This approach does not allow detection of different ESBL-producing strains with the same morphotype at individual time points. The observed change in MLVA-types over time may be caused by an alteration in the relative dominance between the strains. Furthermore, the initial strain may have evolved sufficiently to be perceived as a new genotype over time.

The observed genetic diversity in the fecal isolates has potential implications in an outbreak setting, where attempts to track the spread of individual clones have been customary [6,31,37]. It is relevant to question if such tracking is a reasonable approach, based on the observed diversity among ESBL producing fecal *E*. *coli* strains in individual samples. This diversity has been demonstrated by different methods [15,31,34,38]. A recent study from a high-endemic setting, using whole genome sequencing on 16 *E*. *coli* colonies from each of seven ESBL-positive fecal samples, disclosed a substantial genetic diversity [38]. Similar genetic variations in fecal *E*. *coli* isolates have been observed from patients with UTI in an ESBL-E low-prevalence setting [34]. During an outbreak investigation, the index patient may thus carry a diversity of ESBL producing *E*. *coli* strains. Consequently, cross-transfer should not be ruled out, even if the microbe fingerprints from randomly selected fecal *E*. *coli* colonies differ between patients.

We found a significant association between phylogroups B2/D and prolonged carriage as previously shown (15). Neither MLVA-types, nor single STs, nor any particular CTX-M groups were correlated to prolonged carriage. Our major contributors to phylogroups B2/D are ST131 (n = 33) and ST38 (n = 13). In a previous study, ST131 has been associated with prolonged carriage in a cohort of long term health care facility residents [18], while other studies, like the present, do not find this correlation [15,17]. Our observation support the notion that also other strains than ST131 have potential to colonise the human intestine for a long time. The studies that do not find significant correlation between ST131 and prolonged carriage include samples containing other B2/D strains that could be equally persistent.

The observed differences in risk factors for prolonged carriage in previous studies may be related to several factors. The patient populations are somewhat different, as the other studies mainly included hospitalized patients, there were more men, and the average age was slightly higher. The patients suffered from different conditions; we only included patients with community acquired UTI, while the others also included patients with ESBL-producing bacteria in other clinical specimens. There are also variations in sample size.

In accordance with previous carriage studies [12,15,17], we did not find any correlation between antibiotic use and prolonged ESBL colonisation within the study cohort, neither regarding the antibiotic type or amount, nor the timing between antibiotic treatment and fecal samples (S3 Data). However, there are several studies observing association between previous antibiotic use and *infection* with ESBL-E. The average antibiotic consumption among the study participants was twice as high as in an age- and sex matched cohort from the same region in 2011, i.e. 52.1 DDDs/1,000 patient days in the study group versus 25.5 DDDs/1,000 inhabitants/day in the population group [39]. As the study cohort is selected among patients with UTI, and 12% of participants suffer from recurrent UTI, this is not surprising. Antibiotics create a selective pressure that favours resistant microbes. The ESBL-carrying plasmids also often contain other resistance genes. Thus, antibiotics which do not directly select for ESBL may provide a selective advantage for genetically linked resistance determinants on the same plasmid. Lack of significant correlation between prolonged carriage and the use of antibiotics may well be a type II error due to our limited sample size. Other presumed, but rarely occurring risk factors included in the model, such as more than three household members, male sex and high co-morbidity score, may also be a subject to type II errors.

Surprisingly, there was no association between prolonged carriage and travel to high-endemic countries. Our hypothesis was that travel to high-endemic areas would be a risk factor for acquiring new ESBL-E strains, thereby decreasing the odds of being ESBL-E negative in subsequent samples. We expected that the variety of strains would be greater in the travellers than in others. However, as we have discussed above, the variation in fecal genotypes is large regardless of travel. A different study design would be necessary to clarify whether the observed diversity is caused by plasmid transfer between strains, genomic evolution of strains over time or parallel infection with different strains.

## Conclusion

We observed an overall ESBL-E fecal clearance rate of 56% one year after a UTI caused by an ESBL-producing *E*. *coli* or *K*. *pneumoniae*. UTI caused by E. coli phylogroup B2 or D strains are associated with prolonged fecal ESBL-E carriage. A single negative sample is not sufficient to assume ESBL-clearance. ESBL production can be detected in several fecal *Enterobacteriaceae* species, and in diverse *E*. *coli* genotypes within the same host. When investigating cross-transmission of ESBL producing bacteria in health care institutions, this notion should be taken into account.

## Supporting information

S1 Fig Antibiotic consumption. (TIF)

S1 Data MLVA profiles and MLST. (XLS)

S2 Data Epidemiology and sample results. (SAV)

S3 Data Time-relations between antibiotic treatment and fecal samples. (XLS)

S1 Table STROBE check list. (PDF)

S1 Text Project protocol in Norwegian. (PDF)

S2 Text Project protocol English translation. (PDF)

S3 Text Søraas-2014-High rate of per oral mecillinam treatment failure. (PDF)

S4 Text Søraas-2013-Risk factors for community acquired urinary tract infection. (PDF)
