# Supplementary figures and images for: Fecal carriage of extended spectrum β-lactamase producing Escherichia coli and Klebsiella pneumoniae after urinary tract infection – A three year prospective cohort study
Source: PLoS One. 2017 Mar 7;12(3):e0173510. doi: 10.1371/journal.pone.0173510 (PMC5340397; doi:10.1371/journal.pone.0173510)

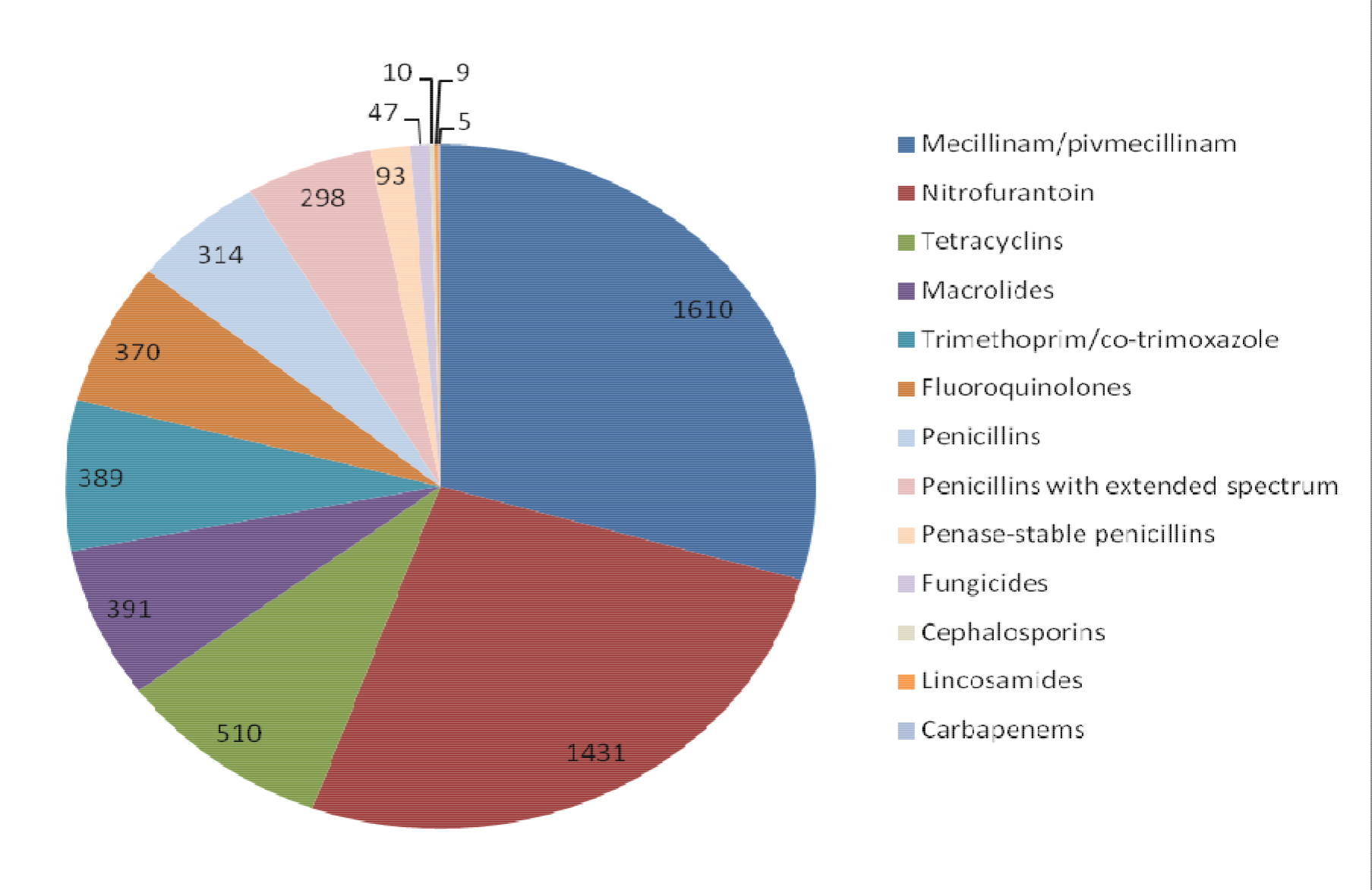

Supplement: S1 Fig — (TIF) [file pone.0173510.s001.tif]
