# Supplementary material for: Fecal carriage of extended spectrum β-lactamase producing Escherichia coli and Klebsiella pneumoniae after urinary tract infection – A three year prospective cohort study
Source: PLoS One. 2017 Mar 7;12(3):e0173510. doi: 10.1371/journal.pone.0173510 (PMC5340397; doi:10.1371/journal.pone.0173510)
Supplement: S1 Text — (PDF) [file pone.0173510.s006.pdf]

VEDLEGG: ESBL STUDIEN - PROTOKOLL

Tittel/arbeidstittel

Ekstendert spektrum beta-laktamase (ESBL) – produserende bakterier. Resistensutbredelse og bærerskap blant polikliniske pasienter og deres nærkontakter

Arbeidstittel

ESBL-bærerskap

Sammendrag

ESBL er plasmidoverførbar resistensmekanisme. Plasmidene har ofte andre resistensgener i tillegg til ESBL. Resistens forårsaket av ESBL er relativt nytt i Norge og det er et økende problem. Enkelte pasienter med ESBL-baktereinfeksjon skal kontaksmitteisoleres ved sykehusinnleggelse og valg av antibakteriell behandling kompliseres ved forekomst av ESBL. Studien er en case control studie som vil se på riskiofaktorer for infeksjon og bærerskap av ESBL. I tillegg vil studien undersøke varigheten av bærerskap av ESBL blant polikliniske pasienter samt deres husstandsmedlemmer.

Prosjektdeltagere

Prosjektleder: Pål A. Jenum, seksjonsoverlege, dr.med.

Prosjektmedarbeidere: Arne Søraas, assistentlege. Annette Onken, overlege, Bioingeniør Professor Arnfinn Sundsfjord, Nasjonalt kompetansesenter for antibiotikaresistens (K-res), Universitetssykehuset i Nord-Norge (UNN).

Professor Inge Olsen ved Institutt for oral biologi ved Universitetet i Oslo.

Introduksjon

Multiresistente gram negative bakterier er et stadig økende problem[14,21]. I Norden har det særlig de siste årene vært en betydelig økning av bakterier med "Extended spectrum betalaktamase" produksjon – såkalte ESBL[3]. Dette er plasmidoverførbar resistens som ofte er koblet med andre resistensmekanismer på samme plasmid. I det siste har man sett at bakterier med denne egenskapen har gått fra å være et problem for pasienter innlagt i sykehus til et problem for den allmenne befolkningen (community aquired ESBL - coESBL) i form av urinveisinfeksjoner som er vanskelige å behandle og i enkelte tilfeller mer alvorlige infeksjoner[10]. Dette fører til forhøyet mortalitet[15], morbiditet og samfunnsøkonomiske tap[5]. Det er grunn til å tro at ESBL egenskapene har begynt å spre seg i normalbefolkningen [6,17]. I Norge er det først og fremst i tarmbakteriene E. coli og K. pneumoniae vi har problemer med ESBL produksjon[17]. Studien er tredelt: Den tar sikte på å studere i) varighet av bærerskap av slike bakterier etter at infeksjon er påvist, ii) undersøke smittsomhet ved å undersøke bærerskap hos nærkontakter/husstandsmedlemmer, samt å studere iii) risikofaktorer for bærerskap av ESBL,

Bakgrunn:

De stadig økende problemene med ESBL internasjonalt har ført til mye forskning på bakterier med denne egenskapen: Man har påvist ESBL bakterier i en rekke materialer[8] blant annet i drikkevann[11], i mat[4,7,19] og i husdyravføring[1,16]. Man vet at bakteriene kan bæres som

tarmbakterier av mennesker og man har mistanke om at bakteriene kan smitte mellom medlemmer i en husholdning[18 12]. Man vet også at ikke alle som blir smittet av bakterier med ESBL får klinisk infeksjon[20]. Det er kjent at multimorbiditet, bruk av antibiotika og innleggelse i sykehus/andre helseinstitusjoner øker risikoen for å få infeksjoner med ESBL[13]. Dessverre gir denne kunnskapen ingen god forklaring på hvorfor forekomsten av coESBL er økende[9]. Man mangler også gode svar på hvilke tiltak som er best for å redusere forekomsten av coESBL**.**

Infeksjoner med coESBL er et potensielt svært alvorlig problem på sykehus fordi ESBL gir resistens mot cefalosporiner som for mange pasienter er førstevalgsantibiotika ved alvorlige infeksjoner. For disse pasientene fører dette til manglende virkning av antibiotika og øket morbiditet og mortalitet[15]. Hvis økningen av coESBL fortsetter kan det føre til at valg av antibiotika på sykehus må endres[9]. Dette vil normalt føre til bruk av mer bredspektrede antibiotika med flere bivirkninger og vesentlig høyere pris.

Problemstilling - formål/mål – hypotese:

To faktorer som er avgjørende for hvordan en epidemi utvikler seg er lengden av bærerskap og hvor smittsom en bærer er. Ingen av disse er godt kartlagte for coESBL.

Studien skal se på:

1. Hvor lenge bærerskap av ESBL i fæces varer ved å undersøke forekomst på forskjellige tidspunkter etter ESBL påvisning.
2. Spredningspotensialet og smitteveier for ESBL ved å undersøke husstandsmedlemmer, inkludert kjæledyr, til pasienter som har hatt ESBL.
3. Risikofaktorer for å få infeksjon og for å bli bærer av ESBL

Studiedesign

Studien er prospektiv. Del 1 og 2 er deskriptiv, mens del 3 er en case control studie. Rekrutteringstiden er satt til den tiden det tar å rekruttere 70 polikliniske pasienter med urinveisinfeksjon med påvist ESBL-produserende bakterier. Dette antas å kunne innfris i løpet av to år. Det vil i tillegg bli valgt ut tre kontroller pasienter per pasient.

1. Bærerskap av bakteriene undersøkes i fæces-penselprøver med to ulike metodikker: 1) Dyrkning på selektive kromogenagarskåler for påvisning av ESBL (cefpodoxim-resistens), 2) PCR for påvisning av gener som koder for ESBL. Studiegruppen vil bli fulgt opp med nye fæcesprøver 5 ganger i ett år.
2. Bærerskap hos husstandsmedlemmer/husdyr av pasienter med påvist ESBL-bakterier undersøkes på samme måte som pasientene.
3. Risikofaktorer for bærerskap undersøkes ved bruk av intervju etter fastlagt spørreskjema.
4. Kontrollgruppen for forekomst vil avlegge en faecesprøve og gjennomgå intervju etter samme spørreskjema.
5. Forekomst av ESBL resistensbærende bakterier undersøkes også i subgingival plakkprøve hos enkelte av pasientene (ikke hos husstandsmedlemmer eller hos kontrollgruppen).

Tilleggsopplysninger om pasientene vil bli innsamlet ved innhenting av opplysninger fra Reseptregisteret samt relevante sykehusjournaler.

Inklusjonskriterier

Gruppe A) Pasienter som i urinprøve har fått påvist E.coli eller Klebsiella pneumoniae bakterier >10.000 bakt/ml med ESBL ved Seksjon for medisinsk mikrobiologi Sykehuset Asker og

Bærum i studieperioden der prøvene er enten 1) tatt utenfor sykehus, sykehjem eller annen helseinstitusjon, eller 2) tatt innleggelsesdagen til helseinstitusjon.

Gruppe B) Husstandsmedlemmer og husdyr til pasientene i Gruppe A

Gruppe C) Polikliniske pasienter som i urinprøve har fått påvist E.coli eller Klebsiella pneumoniae bakterier >10.000 bakt/ml UTEN ESBL ved Mikrobiologisk laboratorium, Sykehuset Asker og Bærum. Det matches to pasienter per Gruppe A pasient. Det matches for kjønn, alder +-5år. De første to som fyller kravene etter diagnose av Gruppe A pasient velges.

Eksklusjonskriterier

1. Pasienter som tidligere har fått påvist ESBL.
2. Pasienter som har vært innlagt i sykehus, sykehjem eller annen helseinstitusjon siste måned.
3. Asylsøkere og immigranter som har vært i Norge <1år.
4. Pasient <18 år gammel eller ikke myndig.

Materiale som skal innhentes og inngå i studien:

1. Bakterieisolatene fra urin som diagnostiseres ha ESBL (Gruppe A). Isolatene fryses.
   (70 isolater)
2. Fæces penselprøver fra gruppe A, tatt 0, 3, 6, 9 og 12 måneder etter påvist ESBL-isolat i urin, en pensel for dyrkning og en pensel for PCR-analyse.
   (350 dyrkningsprøver, 350 PCR-analyser)
3. Fæces penselprøver, en for dyrkning og en for PCR analyse, fra gruppe B og C.
   (Antatt (1,5 B + 2 C) 245 dyrkningsprøver og 245 PCR-analyser
4. Bakterieisolatene fra faeces som diagnostiseres og har ESBL (Gruppe A, B og C). Isolatene fryses.
5. Spørreskjema fra gruppe A, B og C ved inkludering
6. Spørreskjema fra gruppe A ved hver oppfølging.
7. Informasjon fra Resptregisteret om poliklinisk rekvirerte antibiotika i Norge.
8. Dyrkningsmateriale fra subgingivalt plakk hos en del av pasientene i gruppe A.

Flytskjema for innsamling av materiale

1. Ved oppfylt inklusjonskriterium for Gruppe A: Laboratoriet kontakter pasienten direkte ved brev. Det vil bli purret én gang om potensiell deltaker ikke svarer.
   a. Invitasjon om å delta (informasjonsskriv)
   b. Spørreskjema
   c. Samtykkeerklæring
2. Pasientens lege (rekvirenten) får informasjon på svarbrevet at pasienten blir bedt om å delta i bærerstudien.
3. Når samtykke mottas.
   a. Tre pensler (dyrkning og PCR) med transportmedium og hylse, utfylt rekvisisjon, og adressert, frankert returkonvolutt.
   b. Laboratoriet kontakter pasienten per telefon for gjennomgang og utfylling av spørreskjemaet (Gruppe A og C).
4. Laboratoriet plukker ut kontroller (Gruppe C) og sender brev med tilsvarende innhold som under punkt 1.

5. Laboratoriet kontakter angitte husholdskontakter (Gruppe B) ved brev med tilsvarende innhold som under punkt 1.
6. Når samtykkeerklæring er kommet til laboratoriet kontaktes kontroll/husholdskontakt per telefon for gjennomgang og utfylling av spørreskjemaet (Gruppe B) og materiale for å ta prøve sendes ut som under punkt 2a og 2b.
7. Informasjon om resultat av dyrkningsprøvene sendes pasienter som har uttrykt ønske om dette.
8. Opplysninger fra Reseptregisteret om forskrevet antibiotika siste 5 år innhentes for alle deltakere (Gruppa A, B og C) fortløpende etter mottak av samtykkeerklæring.
9. Fra gruppe A innhentes nye fæcesprøver tatt etter 3, 6, 9, 12 måneder. Prøvemateriell og informasjon sendes ut fra laboratoriet på dato for ønsket ny prøve. Mottatte prøver behandles som første prøve.
10. Et begrenset antall pasienter i gruppe A vil bli spurt om å avlegge prøve fra subgingival plakkprøve. Prøven vil tas ved Institutt for oral biologi ved Universitetet i Oslo.

Laboratoriemetode

Primærseleksjon vil foregå på basis av alle laboratoriets motatte urin dyrkningsprøver i studieperioden. Urin inneholdende >10.000 bakterier/ml i renkultur eller med maksimalt to ulike isolater inkluderes. Identifikasjonen av gram-negative staver vil bli gjort med etter laboratoriets rutineprosedyre med kromogenagar (CPS ID 3, BioMerieux) og VITEK-2 (BioMerieux). Resistensbestemmelse utføres med VITEK-2 og ved påvist resistens eller intermediær følsomhet for 3. generasjons cefalosporiner vil undersøkes med ESBL E-test Cefotaxim/Cefotaxim-klavulanat og Ceftazidim/Ceftazidim-klavulanat E-test (AB-biodisk/BioMerieux). Etter behov vil prøvene analyseres ytterligere ved K-res, UNN. Prøver fra subgingivalt plakk dyrkes og analyseres etter rutinene ved Institutt for oral biologi, UIO.

Påvisning og analyse av ESBL fra faeces vil bli gjort ved:

1. Dyrkning på selektive kromogenagarskåler (ChromID ESBL agar, BioMerieux) for påvisning av ESBL-produserende bakterier, Bekreftes ved ESBL E-tester (AB-biodisk/BioMerieux) utført på renkultur etter spredning. Identifikasjon av gram-negative staver: VITEK-2. Resistensbestemmelse: VITEK-2 Bakterieisolater fryses.
2. PCR for påvisning av gener for ESBL (i samarbeid med K-res, UNN). Karakteriseringen av påviste ESBL-typer vil utføres ved K-res, UNN.

Datahåndteringsprosedyrer

Innsamlede data vil bli anonymisert etter at pasienter som ønsker det har fått informasjon om sine resultater. Dataene blir oppbevart og håndtert etter rutinene for databehandling ved Asker og Bærum sykehus.

Statistikk

Deskriptiv (I): Forekomst og varighet av bærerskap av bakterier med ESBL egenskaper for pasienter med urinveisinfeksjon med slik mikrobe.

Case kontroll (I): Risikofaktorer for denne forekomst. Gruppe A mot Gruppe C (kontroll)

Case kontroll (II): Risikofaktorer for å være/bli bærer for pasient i gruppe A blir en. Bærere i gruppe A mot ikke-bærere gruppe A

Deskriptiv (II): Forekomst av assosiert smitte til husstandsmedlemmer.

Case kontroll (II): Risikofaktorer for å være assosiert smittebærer. Bærere i Gruppe B mot ikke-bærere gruppe B.

Relevante statistiske metoder vil bli benyttet for å vurdere risikofaktorer for forekomst og bærerskap.

Gjennomføringsplan

Protokoll, nødvendige godkjenninger og søknad om midler høst 2008

Oppstart: ultimo 2008

Plan for ressursbehov

Delfinansiering av assistentlege lønn for 20 % forskning	150.000
Reagenser, medier og forbruksvarer	100.000

(Kromogenskåler, ID- og resistenskort, E-tester, fryserør, PCR-reagenser: Primere, Prober, Mastermix, prøvepensler, porto etc)

Etiske betraktninger

Målet og studiedesign er i tråd med Helsinki-deklarasjonen. Nødvendige godkjenninger fra Regional etisk komité og Norsk Samfunnsvitenskapelig datatjeneste vil bli innhentet. Deltakelse krever skriftlig informert samtykke fra studiedeltakerne (se vedlagte pasientsamtykkeskjema med informasjonsskriv)

Undersøkelsene som vil bli utført innebærer en liten belastning for deltakerne og vil ikke innebære noen fysisk risiko. Resultatet av undersøkelsen av fæces vil indikere om pasienten er bærer eller ikke-bærer av en bakterie med ESBL. Dette vil som hovedregel ikke ha konsekvenser for deltagernes livsførsel.

Mulige konsekvenser for deltakerne:

1. Pasienter som er syke av eller bærere av ESBL-produserende bakterier kan, ved innleggelse i sykehus eller sykehjem, dersom det er fare for spredning av bakteriene fra pasienten, for eksempel gjennom urin eller avføring (ved inkontinens, stomier eller diaré) bli kontaktsmitteisolert (ved diaré vil kontaktsmitteisolering bli gjort uansett mikrobiologisk funn). For pasienter uten særlig risiko for spredning (de fleste) vil funnet ikke ha noen konsekvenser ved norske sykehus, da det ikke er noen indikasjon for isolering av disse i dag[2].
2. Bærerskap av ESBL kan ha betydning ved senere antibiotikakrevende infeksjoner hos en pasient fordi bakterier med ESBL krever særlig tilpasset antibiotikabehandling[3]. Kunnskap om ESBL-bærerskap kan dermed bidra til at korrekt antibiotika blir gitt på et tidligere tidspunkt og derved bidra til raskere behandlingsrespons og bedre prognose ved infeksjoner[15].

Informasjon om dette gis i informasjonen ved forespørsel om deltakelse

Tillegg av 8.5.2012

Tilleggsmateriale som skal samles inn:
En ekstra rektalpensel fra gruppe A
Et ekstra spørreskjema fra gruppe A.

Reference List

1. NORM RAPPORT SIDE 30. 2006.
Ref Type: Generic

2. Svenske ESBL retningslinjer http://soapimg.icecube.snowfall.se/strama/ESBLdokument%20inkl%20bakgrund.pdf. 2008.
Ref Type: Generic

3. **Fang, H., F. Ataker, G. Hedin, and K. Dornbusch**. 2008. Molecular epidemiology of extended-spectrum beta-lactamases among Escherichia coli isolates collected in a Swedish hospital and its associated health care facilities from 2001 to 2006. J. Clin. Microbiol. **46**:707-712.

4. **Lavilla, S., J. J. Gonzalez-Lopez, E. Miro, A. Dominguez, M. Llagostera, R. M. Bartolome, B. Mirelis, F. Navarro, and G. Prats**. 2008. Dissemination of extended-spectrum beta-lactamase-producing bacteria: the food-borne outbreak lesson. J. Antimicrob. Chemother. **61**:1244-1251.

5. **Lee, S. Y., S. Kotapati, J. L. Kuti, C. H. Nightingale, and D. P. Nicolau**. 2006. Impact of extended-spectrum beta-lactamase-producing Escherichia coli and Klebsiella species on clinical outcomes and hospital costs: a matched cohort study. Infect. Control Hosp. Epidemiol. **27**:1226-1232.

6. **Lewis, J. S., M. Herrera, B. Wickes, J. E. Patterson, and J. H. Jorgensen**. 2007. First report of the emergence of CTX-M-type extended-spectrum beta-lactamases (ESBLs) as the predominant ESBL isolated in a U.S. health care system. Antimicrob. Agents Chemother. **51**:4015-4021.

7. **Machado, E., T. M. Coque, R. Canton, J. C. Sousa, and L. Peixe**. 2008. Antibiotic resistance integrons and extended-spectrum {beta}-lactamases among Enterobacteriaceae isolates recovered from chickens and swine in Portugal. J. Antimicrob. Chemother. **62**:296-302.

8. **Mesa, R. J., V. Blanc, A. R. Blanch, P. Cortes, J. J. Gonzalez, S. Lavilla, E. Miro, M. Muniesa, M. Saco, M. T. Tortola, B. Mirelis, P. Coll, M. Llagostera, G. Prats, and F. Navarro**. 2006. Extended-spectrum beta-lactamase-producing Enterobacteriaceae in different environments (humans, food, animal farms and sewage). J. Antimicrob. Chemother. **58**:211-215.
Ref ID: 13

9. **Pitout, J. D. and K. B. Laupland**. 2008. Extended-spectrum beta-lactamase-producing Enterobacteriaceae: an emerging public-health concern. Lancet Infect. Dis. **8**:159-166.

10. **Pitout, J. D., P. Nordmann, K. B. Laupland, and L. Poirel**. 2005. Emergence of Enterobacteriaceae producing extended-spectrum beta-lactamases (ESBLs) in the community. J. Antimicrob. Chemother. **56**:52-59.

11. **Pokharel, B. M., J. Koirala, R. K. Dahal, S. K. Mishra, P. K. Khadga, and N. R. Tuladhar**. 2006. Multidrug-resistant and extended-spectrum beta-lactamase (ESBL)-producing Salmonella enterica (serotypes Typhi and Paratyphi A) from blood isolates in Nepal: surveillance of resistance and a search for newer alternatives. Int. J. Infect. Dis. **10**:434-438.

12. **Rodriguez-Bano, J., L. Lopez-Cerero, M. D. Navarro, P. D. de Alba, and A. Pascual**. 2008. Faecal carriage of extended-spectrum {beta}-lactamase-producing Escherichia coli: prevalence, risk factors and molecular epidemiology. J. Antimicrob. Chemother.

13. **Rodriguez-Bano, J., M. D. Navarro, L. Romero, L. Martinez-Martinez, M. A. Muniain, E. J. Perea, R. Perez-Cano, and A. Pascual**. 2004. Epidemiology and clinical features of infections caused by extended-spectrum beta-lactamase-producing Escherichia coli in nonhospitalized patients. J. Clin. Microbiol. **42**:1089-1094.

14. **Rossolini, G. M., M. M. D'Andrea, and C. Mugnaioli**. 2008. The spread of CTX-M-type extended-spectrum beta-lactamases. Clin. Microbiol. Infect. **14 Suppl 1**:33-41.

15. **Schwaber, M. J., S. Navon-Venezia, K. S. Kaye, R. Ben-Ami, D. Schwartz, and Y. Carmeli**. 2006. Clinical and economic impact of bacteremia with extended- spectrum-beta-lactamase-producing Enterobacteriaceae. Antimicrob. Agents Chemother. **50**:1257-1262.

16. **Smet, A., A. Martel, D. Persoons, J. Dewulf, M. Heyndrickx, B. Catry, L. Herman, F. Haesebrouck, and P. Butaye**. 2008. Diversity of extended-spectrum beta-lactamases and class C beta-lactamases among cloacal Escherichia coli Isolates in Belgian broiler farms. Antimicrob. Agents Chemother. **52**:1238-1243.

17. **Tofteland, S., B. Haldorsen, K. H. Dahl, G. S. Simonsen, M. Steinbakk, T. R. Walsh, and A. Sundsfjord**. 2007. Effects of phenotype and genotype on methods for detection of extended-spectrum-beta-lactamase-producing clinical isolates of Escherichia coli and Klebsiella pneumoniae in Norway. J. Clin. Microbiol. **45**:199-205.

18. **Valverde, A., F. Grill, T. M. Coque, V. Pintado, F. Baquero, R. Canton, and J. Cobo**. 2008. High rate of Intestinal Colonization with Extended Spectrum {beta}-Lactamases Producing Organisms in Household Contacts of Infected Community Patients. J. Clin. Microbiol.

19. **Warren, R. E., V. M. Ensor, P. O'Neill, V. Butler, J. Taylor, K. Nye, M. Harvey, D. M. Livermore, N. Woodford, and P. M. Hawkey**. 2008. Imported chicken meat as a potential source of quinolone-resistant Escherichia coli producing extended-spectrum beta-lactamases in the UK. J. Antimicrob. Chemother. **61**:504-508.

20. **Warren, R. E., G. Harvey, R. Carr, D. Ward, and A. Doroshenko**. 2008. Control of infections due to extended-spectrum beta-lactamase-producing organisms in hospitals and the community. Clin. Microbiol. Infect. **14 Suppl 1**:124-133.

21. **Woodford, N., M. E. Ward, M. E. Kaufmann, J. Turton, E. J. Fagan, D. James, A. P. Johnson, R. Pike, M. Warner, T. Cheasty, A. Pearson, S. Harry, J. B. Leach, A. Loughrey, J. A. Lowes, R. E. Warren, and D. M. Livermore**. 2004. Community and hospital spread of Escherichia coli producing CTX-M extended-spectrum beta-lactamases in the UK. J. Antimicrob. Chemother. **54**:735-743.
