# Supplementary material for: Fecal carriage of extended spectrum β-lactamase producing Escherichia coli and Klebsiella pneumoniae after urinary tract infection – A three year prospective cohort study
Source: PLoS One. 2017 Mar 7;12(3):e0173510. doi: 10.1371/journal.pone.0173510 (PMC5340397; doi:10.1371/journal.pone.0173510)
Supplement: S2 Text — (PDF) [file pone.0173510.s007.pdf]

ANNEX: ESBL STUDY - PROTOCOL

Title / working title

Extended-spectrum beta-lactamase (ESBL)-producing bacteria.
Resistance distribution and carriage among outpatients and their close contacts

Working title

ESBL carriage

Summary

ESBL is transmitted by plasmids. Plasmids often contain other antimicrobial resistance genes besides ESBL. Resistance caused by ESBL is a relatively new phenomenon in Norway, but it is a growing problem. Some patients with ESBL are isolated under contact precautions when hospitalized, and antibiotic treatment is complicated by the occurrence of ESBL.
This is a case control study that will seek to define risk factors for infection and carriage of ESBL. In addition, we will describe ESBL-carriage duration among outpatients and their household members.

Project participants

Project Director: Pål A. Jenum, chief physician, MD, Ph.D.

Researchers: Arne Søraas, resident MD. Annette Onken, senior physician MD, Professor Arnfinn Sundsfjord, National Centre for antibiotic resistance (K-res), University Hospital of North Norway (UNN).

Professor Ingar Olsen, Department of Oral Biology, University of Oslo.

Introduction

Multiresistant gram negative bacteria are an increasing problem [14,21]. In the Nordic countries there has recently been a significant increase of bacteria with "Extended spectrum beta-lactamase" production - so-called ESBL [3]. This is plasmid mediated antimicrobial resistance that is often connected with other resistance mechanisms on the same plasmid. Bacteria with these plasmids have gone from being a problem in hospitalized patients, to being a problem in the general population (community acquired ESBL - coESBL), both in the form of urinary tract infections that are difficult to treat, and in some cases in the form of more severe infections [10]. This leads to increased mortality [15], morbidity and economic losses [5]. There is reason to believe that ESBL has begun to spread in the general population [6,17]. In Norway it is primarily in E. coli and K. pneumoniae we have problems with ESBL-production [17].

The study has three aims: i) to study duration of ESBL-carriage after urinary tract infection, ii) to investigate the infectivity by examining carriers with close contacts / household members, iii) to study risk factors for ESBL-carriage,

Background:

The ever increasing problems with ESBL internationally have led to much research: ESBL producing bacteria have been found in a variety of materials including [8] drinking water [11], food [4,7,19] and animal faeces [1,16]. We know that these bacteria colonize the human gut, and it is suspected that the bacteria are transferred between household members [18] [12]. We also know that not everyone who becomes colonized with ESBL producing bacteria get a clinical infection [20]. It is known that multimorbidity, antibiotic use and hospitalization/other contact with health care institutions increases the risk of getting ESBL infections [13]. Unfortunately, this knowledge gives no good explanation to why the incidence of coESBL is growing [9]. Neither do we know what measures are most efficient in reducing the incidence of coESBL.

Infections caused by coESBL are a potentially very serious problem in hospitals, because ESBL confer resistance to cephalosporins which are first choice antibiotics for serious infections in many patients. For the affected patients, ESBL leads to failure of antibiotic therapy and increased morbidity and mortality [15]. If the increase in coESBL continues, the recommendations on empirical antibiotic treatment in hospitals must be changed [9]. This would normally lead to the use of more broad-spectrum antibiotics with more side effects and considerably higher price.

Issues - purpose / goal - hypothesis:

Two factors are crucial for how the epidemic is evolving; the carriage duration and the contagiousness of the carrier. None of these factors are well-known for coESBL.

We want to study:

1. The duration of ESBL fecal carriage, by examining the prevalence at different time points after ESBL detection.
2. Proliferation potential and transmission routes for ESBL by examining household members, including pets, to patients who have had ESBL.
3. Risk factors for getting an infection, and risk factors for becoming a fecal carrier of ESBL .

Study design

The study is prospective. Part 1 and 2 are descriptive, while part 3 is a case-control study. The enrolment duration is set to the time required to recruit 70 outpatients with urinary tract infection caused by ESBL-producing bacteria. This is believed to be achieved within two years. Additionally two control patients per ESBL-patient will be included.

1. ESBL detection from faecal swabs will be achieved by two different methods:
 1) Culture on selective chrome agar plates designed for detection of ESBL (Cefpodoxime resistance),
 2) PCR for detection of ESBL encoding genes. The study group will be followed with new fecal samples five times during one year.
2. ESBL-carriage in household members / pets of patients with proven ESBL infection will be examined in the same way as for patients.

3. Risk factors for ESBL-carriage will be examined through interviews by use of an established questionnaire.
4. The control group will deliver one faecal sample and undergo an interview using the same questionnaire.
5. Prevalence of ESBL producing bacteria in subgingival plaque samples from some of the patients (not from household members or from the control group) will also be examined.

Additional information regarding the patients will be collected from the Norwegian Prescription Database and from relevant medical records.

Inclusion criteria

Group A) Patients who have delivered a urine sample containing >10,000 cfu/ml of ESBL-producing E.coli or Klebsiella pneumoniae to the Department of medical microbiology at Asker and Baerum hospital during the study period. The urine samples must either 1) have been taken outside the hospital, nursing home or other health care institution, or 2) have been taken on the day of admission to a health care institution.

Group B) Household members and pets of patients in Group A

Group C) Outpatients with urine samples containing >10,000 cfu/ml E.coli or Klebsiella pneumoniae WITHOUT ESBL at the Department of medical microbiology at Asker and Bærum Hospital. We will match two patients per group A patient. Matching will be done by sex and age + -5 years. The first two cases that appear after a patient in Group A are chosen.

Exclusion criteria

1. Patients who previously have been diagnozed with ESBL.
2. Patients who have been admitted to hospital, nursing home or other health care institution within the last month.
3. Asylum seekers and immigrants who have been in Norway <1year.
4. Patients <18 years old or not able to give informed consent.

Material to be collected and included in the study:

1. ESBL-producing bacterial isolates from urine (Group A). The isolates will be frozen for storage (70 isolates).
2. Faecal swabs from Group A, taken 0, 3, 6, 9 and 12 months after the ESBL-isolate was diagnosed in urine, a swab for cultivation and a swab for PCR analysis. (350 culture samples, 350 PCR assays).
3. Faecal swabs, one for cultivation and one for PCR analysis, from group B and C.
(Estimated (1.5 B + 2 C) 2 45 culture samples and 2:45 PCR analyzes
4. ESBL-producing bacterial isolates from faeces (Group A, B and C). The isolates are frozen for storage.
5. Questionnaire from Groups A, B and C at inclusion
6. Questionnaire from group A at each follow-up.

7. Information from the National Prescription Database concerning antibiotic prescription to the participants.

8. Culture material from subgingival plaques of a portion of the patients in group A.

Flowchart for material collection

1. If inclusion criteria for Group A are fulfilled: The laboratory contacts the patient directly by letter. A reminder is sent if a potential participant does not respond.
    a. Invitation to participate (information sheet)
    b. Questionnaire
    c. Consent Statement
2. The patient's physician (who has asked for the primary urine culture analysis) is informed that the patient is asked to participate in the study in the reply letter from the laboratory.
3. Upon returning a signed consent form, the participants receive by mail
    a. Sample swabs (culture and PCR) with transport medium, completed requisition, and addressed, stamped return envelope.
    b. The laboratory contacts the patient via telephone for review and to complete the questionnaire (Group A and C).
4. The laboratory select controls (Group C) and send letters with similar content as in paragraph 1.
5. The laboratory contacts specified household members (Group B) by letter with similar content as described in paragraph 1.
6. When written statement of consent has been returned, the laboratory contacts the control / household member by phone for review and completion of the questionnaire (Group B), and sends out sample kits to sample materials as described in subparagraph 2a and 2b.
7. Information about the result of the cultured sample is sent to patients who have expressed a desire for this.
8. Information from the Norwegian Prescription Database on prescribed antibiotics past 5 years will be obtained for all participants (group A, B and C) consecutively after receiving the consent form.
9. From group A new faecal samples will be obtained after 3, 6, 9, 12 months. Sample kots and information is sent out from the laboratory on the desired date of new testing. Subsequent samples will be processed as the first.
10. A limited number of patients in group A will be asked to deliver a subgingival plaque sample. The sampling will take place at the Department of Oral Biology at the University of Oslo.

Laboratory methods

Primary selection will take place on the basis of all submitted urine culture samples to our laboratory in the study period. Urine containing> 10,000 cfu / ml in pure culture or with a maximum of two morphologically different isolates will be included. The identification of gram-negative rods follows our laboratory's routine procedure with chrome agar (CPS ID 3, BioMerieux) and VITEK-2 (BioMerieux). Susceptibility testing is performed with VITEK-2 and if non-susceptibility to 3rd generation cephalosporins are detected, we will test for ESBL by cefotaxime / cefotaxime clavulanate and Ceftazidim / Ceftazidim clavulanate E-tests (AB-biodisk / BioMerieux). As required samples will be analyzed further by the Reference centre for microbial resistance, UNN. Samples from subgingivalt plaque will be cultured and analyzed by the procedures of the Department of Oral Biology, University of Oslo.

Detection and analysis of ESBL from faeces:

1. Cultivation on selective chromogenic agar (ChromID ESBL agar, BioMerieux) for detection of ESBL-producing bacteria, confirmed by ESBL E-test (AB-biodisk / BioMerieux) performed on pure culture after subcultivation. Identification of gram-negative rods: VITEK-2. Susceptibility testing: VITEK-2. Freezing of isolates.
2. PCR for detection of ESBL-encoding genes (in collaboration with UNN). The characterization of different ESBL-types will be performed by K-res, UNN. Agreed samarebeid with K-res Professor Sundsfjord)

Data Handling Procedures

Collected data will be anonymised when the patients who want to has received information about their results. The data will be stored and handled according to the routine for research data at Asker and Bærum hospital.

Statistics

Descriptive (I): Prevalence and duration of ESBL-producing Enterobacteriaceae in patients with urinary tract infection caused by .such microbes.

Case control (I): Risk factors for urinary tract infection with ESBL-producing microbes. Group A compared with Group C (control)

Case control (II): Risk factors to be / become a faecal carrier for patients in group A. Faecal carriers in Group A versus faecal non-carriers in Group A.

Descriptive (II): Incidence of associated carriage in household members..

Case control (III): Risk factors of being an associated carrier. Carriers in Group B versus non-carriers Group B.

Relevant statistical methods will be used to examine potential risk factors for infection and carriage.

Implementation Plan

Protocol, necessary approvals and applications for funds fall 2008

Startup: ultimo 2008

Plan resource needs

Co-financed by resident salaries for 20 % Research	150.000
Reagents, media and consumables	100,000

(Chrome agar plates, ID and susceptibility cards, E-tests, tubes, PCR reagents: Primers, Probes, Mastermix, swabs, postage etc)

Ethical considerations

Objective and study design is in line with the Helsinki-declaration. Necessary approvals from the Regional Ethics Committee and the Norwegian Social Science Data Services will be obtained. Participation requires written informed consent from study participants (see attached patient consent form information sheet).

The investigations that will be carried out involve a small burden for the participants and will not involve any physical risk. The result of the faecal tests will indicate whether the patient is a carrier or non-carrier of bacteria with ESBL. This will generally not have an impact on the participants' behaviour.

Possible consequences for participants:

1. Patients who are infected with or carriers of ESBL-producing bacteria may be isolated under contact precautions upon admission to a hospital or nursing home. This is if they have individual risk factors of increased transmission of gram-negative rods, e.g. incontinence, stoma or diarrhoea. For patients without individual risk factors, knowledge of their ESBL faecal carriage status will have no consequences in Norwegian hospitals, as ESBL-carriers generally do not require contact precautions for the time being [2.].
2. Knowledge of ESBL-carriage status may influence future antibiotic therapy, because ESBL-producing bacteria require especially adapted antibiotic treatment [3.] Knowledge of ESBL-carriage status may thus lead to correct empirical antibiotic therapy at an early stage, and thereby contribute to faster treatment response and better infection prognosis [15.].

These ethical considerations are presented to the patients when they are requested to participate in the study.

Addition May 8th 2012

Material to be collected and included in the study:

One additional faecal swab for cultivation from Group A.

One additional questionnaire from Group A.

Reference List

1. NORM REPORT PAGE 30 2006.
Ref Type: Generic

2. Swedish ESBL guidelines http://soapimg.icecube.snowfall.se/strama/ESBLdokument%20inkl%20bakgrund.pdf. 2008.
Ref Type: Generic

3. **Fang, H., F. Atak, G. Hedin, and K. Dornbusch.** 2008. Molecular epidemiology of extended-spectrum beta-lactamases Among Escherichia coli isolates Collected in a Swedish hospital and its Associated Healthcare Facilities from 2001 Thurs 2006. J. Clin. Microbiol. **46:** 707-712.

4. **Lavilla, S., JJ Gonzalez-Lopez, E. Miro, A. Dominguez, M. Llagostera, RM Bartolome, B. Mirelis, F. Navarro and G. Prats.** 2008. Dissemination of extended-spectrum beta-lactamase-producing bacteria, the food-borne outbreak lesson. J. Antimicrob. Chemother. **61:** 1244-1251.

5. **Lee, SY, S. Kotapati, JL Kuti, CH Nightingale, and DP Nicolau.** 2006. Impact of extended-spectrum beta-lactamase-producing Escherichia coli and Klebsiella species on clinical outcomes and hospital costs: a matched cohort study. Infect. Control Hosp. Epidemiol. **27:** 1226-1232.

6. **Lewis, JS, M. Herrera, B. Wickes, JE Patterson, and JH Jorgensen.** 2007. First report of the emergence of CTX-M-type extended-spectrum beta-lactamases (ESBLs) as the predominant ESBL isolated in a US healthcare system. Antimicrob. Agents Chemother. **51:** 4015-4021.

7. **Machado E., TM Coque, R. Canton, JC Sousa and L. Peixe.** 2008. Antibiotic resistance integrons and extended-spectrum {beta} -lactamases Among Enterobacteriaceae isolates Recovered from chickens and swine in Portugal. J. Antimicrob. Chemother. **62:** 296-302.

8. **Mesa, RJ, V. Blanc, AR Blanch, P. Cortes, JJ Gonzalez, S. Lavilla, E. Miro, M. Muniesa, M. Saco, MT Tortola, B. Mirelis, P. Coll, M. Llagostera, G . Prats, and F. Navarro.** 2006. Extended-spectrum beta-lactamase-producing Enterobacteriaceae in different environments (humans, food, animal farms and sewage). J. Antimicrob. Chemother. **58:** 211-215.
Ref ID: 13

9. **Pitout, JD and KB Laupland.** 2008. Extended-spectrum beta-lactamase-producing Enterobacteriaceae: an emerging public-health concern. Lancet Infect. Dis. **8:** 159-166.

10. **Pitout, JD, P. Smith, KB Laupland, and L. POIREL.** 2005. Emergence of Enterobacteriaceae producing extended-spectrum beta-lactamases (ESBLs) in the community. J. Antimicrob. Chemother. **56:** 52-59.

11. **Pokharel, BM, J. Koirala, RK Dahal, SK Mishra, PK Khadgar, and NR Tuladhar.** 2006. Multi Drug-resistant and extended-spectrum beta-lactamase (ESBL) -producing Salmonella enterica (serotypes typhi and paratyphi A) from blood isolates in Nepal: surveillance of resistance and a search for alternatives Reviews newer. Int. J. Infect. Dis. **10:** 434-438. 12. **Rodriguez-Bano, J., L. Lopez-Cerero, MD Navarro, PD de Alba, and A. Pascual.** 2008. faecal carriage of extended-spectrum {beta} -lactamase-producing Escherichia coli: prevalence, risk factors and molecular epidemiology. J. Antimicrob. Chemother.

13. **Rodriguez-Bano, J., MD Navarro, L. Romero, L. Martinez-Martinez, MA Muniain, EJ Perea, R. Perez-Cano, and A. Pascual.** 2004. Epidemiology and clinical features of Infection caused by extended-spectrum beta-lactamase-producing Escherichia coli in nonhospitalized Patients. J. Clin. Microbiol. **42:** 1089-1094.

14. **Rossolini, GM, MM D'Andrea, and C. Mugnaioli.** 2008. The spread of CTX-M-type extended-spectrum beta-lactamases. Clin. Microbiol. Infect. **14 Suppl 1:** 33-41.

15. **Schwaber, MJ, S. Navona Venice, KS Kaye, R. Ben-Ami, D. Schwartz, and Y. Carmeli.** 2006. Clinical and economic impact of bacteremia with extended- spectrum beta-lactamase-producing Enterobacteriaceae. Antimicrob. Agents Chemother. **50:** 1257-1262.

16. **Smet, A., A. Martel, D. Persoons, J. Dewulf, M. HEYNDRICKX, B. Catry, L. Herman, F. Haesebrouck, and P. Butaye.** 2008. Diversity of extended-spectrum beta-lactamases and class C beta-lactamases Among cloacal Escherichia coli isolates in Belgian broiler farms. Antimicrob. Agents Chemother. **52:** 1238-1243.

17. **Tofteland, S., B. Haldorsen, KH Dahl, GS Simonsen, M. Steinbakk, TR Walsh, and A. Sundsfjord.** 2007. Effects of phenotype and genotype on methods for detection of extended-spectrum beta-lactamase-producing clinical isolates of Escherichia coli and Klebsiella pneumoniae in Norway. J. Clin. Microbiol. **45:** 199-205.

18. **Valverde, A., F. Grill, TM Coque, V. Pintado, F. Baquero, R. Canton, and J. Cobo.** 2008. High rate of Intestinal Colonization with Extended Spectrum {beta} -Lactamases Producing Organisms in Household Contacts of Infected Patients Community. J. Clin. Microbiol.

19. **Warren, RE, VM Ensor, P. O'Neill, V. Butler, J. Taylor, K. New, M. Harvey, DM Livermore, N. Woodford, and PM Hawkey.** 2008. Imported chicken meat as a potential source of quinolone-resistant Escherichia coli producing extended-spectrum beta-lactamases in the UK. J. Antimicrob. Chemother. **61:** 504-508.

20. **Warren, RE, G. Harvey, R. Carr, D. Ward, and A. Doroshenko.** 2008. Control of Infection due two extended-spectrum beta-lactamase-producing organisms in hospitals and the community. Clin. Microbiol. Infect. **14 Suppl 1:** 124-133.

21. **Woodford, N., ME Ward, ME Kaufmann, J. Turton, EJ Fagan, D. James, AP Johnson, R. Pike, M. Warner, T. Cheasty, A. Pearson, S. Harry, JB Leach, A. Loughrey, JA Lowes, RE Warren, and DM Livermore.** 2004. Community and hospital spread of Escherichia coli producing CTX-M extended-spectrum beta-lactamases in the UK. J. Antimicrob. Chemother. **54:** 735-743.
