# Supplementary material for: Fecal carriage of extended spectrum β-lactamase producing Escherichia coli and Klebsiella pneumoniae after urinary tract infection – A three year prospective cohort study
Source: PLoS One. 2017 Mar 7;12(3):e0173510. doi: 10.1371/journal.pone.0173510 (PMC5340397; doi:10.1371/journal.pone.0173510)
Supplement: S3 Text — (PDF) [file pone.0173510.s008.pdf]

High Rate of Per Oral Mecillinam Treatment Failure in Community-Acquired Urinary Tract Infections Caused by ESBL-Producing *Escherichia coli*

Arne Søraas[1]*, Arnfinn Sundsfjord[2], Silje Bakken Jørgensen[1], Knut Liestøl[3], Pål A. Jenum[1,4]

1 Department of Medical Microbiology, Vestre Viken Hospital Trust, Bærum, Norway, **2** Department of Microbiology and Infection Control, Reference Centre for Detection of Antimicrobial Resistance, University Hospital of North Norway, Tromsø, Norway, and Department of Medical Biology, Research Group for Host-Microbe Interactions, Faculty of Health Sciences, University of Tromsø, Tromsø, Norway, **3** Department of Informatics, University of Oslo, Oslo, Norway, **4** Department of Clinical Medicine, Faculty of Medicine, University of Oslo, Oslo, Norway

Abstract

A population-based study was performed to investigate the efficacy of mecillinam treatment of community-acquired urinary tract infections (CA-UTI) caused by extended-spectrum β-lactamase (ESBL) producing *Escherichia coli.* The study was conducted in South-Eastern Norway. Data from patients with CA-UTI caused by ESBL-producing and non-producing (random controls) *E. coli* were collected through interviews, questionnaires, medical records and the Norwegian Prescription Database. Treatment failure was defined as a new antibiotic prescription appropriate for UTI prescribed within two weeks after the initial antimicrobial therapy. Multivariable logistic regression analysis was performed to identify treatment agents and patient- or bacterial traits associated with treatment failure. A total of 343 patients (mean age 59) were included, of which 158 (46%) were treated with mecillinam. Eighty-one patients (24%, mean age 54) had infections caused by ESBL producing *E. coli,* and 41 of these patients (51%) received mecillinam as the primary treatment. Mecillinam treatment failure was observed in 18 (44%) of patients infected by ESBL-producing strains and in 16 (14%) of patients with a CA-UTI caused by ESBL non-producing strains. Multivariable analysis showed that ESBL status (odds ratio (OR) 3.2, 95% confidence interval (CI) 1.3–7.8, $p = 0.009$) and increased MIC of mecillinam (OR 2.0 for each doubling value of MIC, CI 1.4–3.0, $p < 0.001$) were independently associated with mecillinam treatment failure. This study showed a high rate of mecillinam treatment failure in CA-UTIs caused by ESBL producing *E. coli*. The high failure rate could not be explained by the increased MIC of mecillinam alone. Further studies addressing the use of mecillinam against ESBL-producing *E. coli,* with emphasis on optimal dosing and combination therapy with β-lactamase inhibitors, are warranted.

Citation: Søraas A, Sundsfjord A, Jørgensen SB, Liestøl K, Jenum PA (2014) High Rate of Per Oral Mecillinam Treatment Failure in Community-Acquired Urinary Tract Infections Caused by ESBL-Producing *Escherichia coli*. PLoS ONE 9(1): e85889. doi:10.1371/journal.pone.0085889

Editor: Dongsheng Zhou, Beijing Institute of Microbiology and Epidemiology, China

Received October 2, 2013; **Accepted** December 6, 2013; **Published** January 15, 2014

Copyright: © 2014 Søraas et al. This is an open-access article distributed under the terms of the Creative Commons Attribution License, which permits unrestricted use, distribution, and reproduction in any medium, provided the original author and source are credited.

Funding: The study were funded by the South Eastern Norway Health Authority. The funder had no role in study design, data collection and analysis, decision to publish, or preparation of the manuscript.

Competing Interests: The authors have declared that no competing interests exist.

* E-mail: arne@meg.no

Introduction

Escherichia coli is the most common cause of community-acquired urinary tract infection (CA-UTI). The worldwide dissemination of multidrug resistant CTX-M extended spectrum β-lactamase (ESBL)-producing *E. coli* has significantly limited the oral treatment options for CA-UTI [1]. Mecillinam is an amidinopenicillin with selective activity against Gram-negative bacteria and *Enterobacteriaceae* in particular. It is widely used in the Scandinavian countries, but the guidelines regarding dosage varies between countries with 200 mg thrice daily (TID) usually prescribed in Norway. In vitro data suggest that mecillinam has a favourable stability to β-lactamase hydrolysis compared with other penicillins [2]. International treatment guidelines endorse the use of mecillinam with an A1-grading of recommendation as a first choice treatment for uncomplicated lower urinary tract infection in women [3]. Mecillinam can be administered per os as a prodrug, the pivaloyloxymethyl ester pivmecillinam, which after absorption is converted to the antibacterial active mecillinam [4]. Mecillinam has been shown to exert a minor ecological impact on the human commensal flora [5,6]. The favourable ecological profile is also underlined by the observed stable and low (<2%) rate of resistance to mecillinam in uropathogenic *E. coli* in repeated international surveys as well as in Scandinavian countries with a widespread use of pivmecillinam over many years [7,8].

In vitro antimicrobial susceptibility tests have provided favourable results for mecillinam against CTX-M producing *E. coli* [9–12]. However, a clavulanate reversible inoculum dependent effect that significantly increases the minimum inhibitory concentration (MIC) for mecillinam in ESBL-producing *E. coli* compared to non-producers has been reported [13,14]. Titelman et. al also found a low bacteriological cure rate (two of eight patients) in a recent study and these notions underline the need for studies addressing the clinical efficacy of mecillinam in CA-UTI caused by ESBL producing *Enterobacteriaceae* [10]. To our knowledge only case-report studies have been reported so far [10,15].

In this population-based study we aimed to prospectively examine the clinical efficacy of mecillinam in the treatment of CA-UTI caused by ESBL-producing *E. coli* compared to non-ESBL-producing *E. coli*.

Patients and Methods

Ethics Statement

The study was approved by the Regional Committee for Medical and Health Research Ethics – South East ("REC South East"), following the Declaration of Helsinki principles (reference number: 2009/2037 BS-08901b). It is registered in ClinicalTrials.gov (Identifier: NCT01838213).

Setting and Design

This study was part of an investigation of CA-UTI and faecal carriage of ESBL-producing bacteria conducted in Norway at the Department of Medical Microbiology, Vestre Viken Hospital Trust, between February 2009 and May 2012 [16]. The hospital trust serves approximately 450.000 inhabitants and is situated in a mixed urban, suburban and rural area in the South-Eastern part of the country. Our two laboratories analyze in- and outpatient samples from this area.

Patients with any type of CA-UTI caused by ESBL-producing or non-ESBL producing *E. coli* and receiving empirical treatment were included in the study. Data on treatment outcome were obtained and possible associations between outcome and mecillinam treatment, ESBL-status and other variables were investigated.

Participants

The eligible population constituted all patients ≥18 years old with a urine culture yielding *E. coli* >10,000 CFU/ml. We excluded patients who: i) had not been empirically treated (i.e., did not collect an antimicrobial agent appropriate for UTI (trimethoprim, trimethoprim-sulfamethoxazole, ciprofloxacin, ofloxacin, nitrofurantoin, pivmecillinam, amoxicillin or cephalexin) at a Norwegian pharmacy at the index date (fosfomycin and amoxicillin/clavulanate are not available in Norway), ii) had lived in Norway for <1 year, iii) were unable to answer the questionnaire, iv) had previously diagnosed infection caused by ESBL-producing bacteria, or v) had health care associated UTI (i.e., had been hospitalized or residing in a nursing home for >24 hours during the last 31 days).

Procedures for inclusion of participants and data collection have been described earlier [16]. In brief, participation required a written consent; all eligible patients with an ESBL-producing *E. coli* were invited to participate. For each patient with an ESBL-producing *E. coli* invited, 2–5 patients with non-ESBL *E. coli* urine isolates during the same time period were randomly selected (Excel® randomization, Microsoft, Redmond, WA) and invited to participate. Participants answered standardized questionnaires which included queries about the current UTI, previous UTIs, contact with the health care system, catheter use and adherence to antibiotic prescriptions. Detailed data about antimicrobial drugs dispensed were collected from The Norwegian Prescription Database and from medical records [17]. To quantify the number of UTIs for each patient in the preceding year, the number of prescriptions of three antimicrobial agents–trimethoprim, mecillinam, and nitrofurantoin–were counted in individual patients. In Norway, these agents are first choices for UTI treatment and are not prescribed for other infections.

Microbiological Data and Antibiotic Susceptibility

Urine cultivation and bacterial identification were performed using ChromID CPS3 agar and the VITEK-2 system (both BioMerieux, Marcy l'Etoile, France). Antimicrobial susceptibility testing and interpretations including ESBL screening were performed using VITEK-2 (AST- N029, N122 or N209 card) which reports MIC of mecillinam in categories ≤1, 2, 4, 8, 16, 32 and ≥64 mg/L based on measurements in wells with mecillinam concentrations of 1, 3, 8 and 32 mg/L. All isolates resistant to cefpodoxime, cefotaxime or ceftazidime were selected for confirmatory ESBL testing using the Etest gradient system (AB-Biodisk, BioMerieux). Clinical breakpoint interpretations were according to EUCAST. The breakpoint for resistance for mecillinam in *E. coli* was >8 mg/L during the study period [18].

Molecular Detection of ESBL

ESBL genotype analysis was performed using PCR for $bla_{\text{CTX-M}}$ detection and group assignment, as described [19]. Detection of bla_{TEM} and bla_{SHV} was performed on ESBL-positive isolates negative for $bla_{\text{CTX-M}}$ using consensus PCR followed by DNA sequencing [20].

Treatment Outcome Measures

Two different treatment failure measures were obtained and compared: i) a patient receiving a second antibiotic prescription appropriate for UTI (same antibiotics as in inclusion) within day 1–14 after the index date [21–23] ii) a patient reporting not to have been subjectively cured within 14 days after initial treatment.

Statistical Analysis

The statistical analyses were conducted using PASW statistics software, version 19.0 (IBM SPSS, Chicago, IL). Univariate analyses were performed using logistic regression, Pearson chi square, Fisher's exact test, Student's t-test or the Mann-Whitney U-test as appropriate. The association between variables and treatment failure was quantified by odds ratio (OR) with 95% confidence interval (CI). Variables with a $p<0.15$ were considered candidates for the multivariable model. A manual backward stepwise elimination procedure using multivariable logistic regression was performed to identify independent risk factors for treatment failure. Multivariable analyses were preceded by estimation of correlation between risk factors and followed by testing of all initial variables added to the final model. All p-values were two-tailed, and a p-value of <0.05 was considered significant. The two outcome measures were compared using Cohens kappa.

Results

A total of 478 (1.5%) of approximately 32.000 urine samples analysed during the inclusion period yielded an ESBL-producing *E. coli*. Of these 478 samples, 231 (48%) were from ineligible patients (mostly because of earlier ESBL and contact with the health care system) and 247 (52%) were from eligible patients. Of these, 132 (53%) consented to participate, but 49 (37%) had not received an antimicrobial at index date and 2 were ineligible for other reasons leaving 81 participants. Among 1330 randomly selected patients with non-ESBL UTI, 453 (34%) consented to participate. Of these, 185 (41%) had not received an antimicrobial drug at index date and six were ineligible for other reasons leaving 262 participants with non-ESBL UTI.

Participants

The study population had a mean age of 59 years (range 18–93 years), which was comparable to that of all patients invited to participate (62 years). In total, 87% of the participants were female. The mean age of patients with an ESBL-producing *E. coli* was 54 years (range 18–92 years), which was significantly younger than patients with a non-ESBL-producing strain (61 years). There were no significant differences between patients with ESBL-positive or ESBL-negative UTI in relation to gender, prescribed

treatment (type or duration) or number of urinary tract infections during the past year.

Antibiotic Susceptibility

An overall higher prevalence of antimicrobial-resistance was detected in ESBL-producing strains than in non-producers (Table 1). The MIC of mecillinam in ESBL-producing strains was higher than in non-ESBL producing strains (2 mg/L (inter-quartile range (IQR) 0 to 4 mg/L) vs. ≤1 mg/L (IQR ≤1 to ≤1 mg/L), p<0.001).

Molecular Detection of ESBL

PCR and sequence analyses showed that 68%, 28%, and 2.5% of the ESBL isolates belonged to the CTX-M group 1, CTX-M group 9 and SHV group 5/12, respectively. One ESBL-isolate was not available for ESBL-typing. TEM-type ESBLs were not detected.

Treatment Outcome Measures

Information on repeat prescriptions (interpreted as treatment failure) were available from the Norwegian Prescription Database and medical records for all participants (n = 343). Clinical data to assess the clinical outcome were available for 251 patients (73%) only. The participants with missing information on the clinical outcome were evenly distributed between the ESBL-positive and ESBL-negative groups. There was substantial agreement between the two outcome measures with Cohen's kappa = 0.70 and congruent results in 219 (87%) of cases evaluable with both methods [24]. Due to the completeness of data, results based on the prescription registry (repeat prescriptions) will be presented henceforth.

Treatment Outcome

In total, 101 (29%) treatment failures as determined by repeat prescriptions were recorded, of which 73 (72%) occurred within the first seven days after initiation of treatment. The treatment failure rate was higher among patients with an ESBL-positive strain (53%) than an ESBL-negative strain (22%) (p<0.001). There were no significant differences in treatment outcome between the different ESBL genotypes.

Treatment outcomes were compared between patients treated with mecillinam (mecillinam-group) and those treated with other antimicrobials (non-mecillinam group). The two groups were similar with regard to background characteristics with the exception of gender and prescribed dose. Females were given mecillinam treatment more frequently than males (49% vs. 23%, respectively, p = 0.001). The mean dose of antimicrobial agent dispensed for the actual UTI was 8.3 defined daily doses (DDD) in the mecillinam group as compared to 6.1 DDD in the non-mecillinam group (p<0.001). Approximately 75% of the patients received a prescription for seven days or more as judged from the number of DDDs. Self-reported compliance with prescribed antibiotics exceeded 90% in both treatment groups.

In the mecillinam treatment group the rate of treatment failure among patients with ESBL-producing strains was 44% vs. 14% for patients with non-ESBL producers (Figure 1). Age, the strain's ESBL status, MIC of mecillinam and overall resistance profile were associated with treatment failure (Table 2). Treatment failed in all four patients with strains that were *in vitro* resistant to mecillinam (3 ESBL-positive strains and 1 ESBL-negative strain). In contrast, we observed a much lower rate of treatment failure (20%) in patients (n = 15) with ESBL-producing strains with a low mecillinam MIC (≤1 mg/L).

In the non-mecillinam treatment group the overall prevalence of treatment failure among patients with and without ESBL-producing strains was 63% and 29%, respectively. Furthermore, the prevalence of treatment failure was 85% and 16% in patients who received an antimicrobial for which their strain was *in vitro* resistant or non-resistant, respectively. In vitro resistance to the dispensed antimicrobial agent (i.e., inappropriate initial treatment), ESBL status and overall resistance profile were associated with treatment failure (Table 3).

Multivarible Analysis

Results from the multivariable analysis are presented in Table 4. The multivariable analyses were performed separately on each of the two treatment groups.

Mecillinam treatment group. The ESBL status and the strain's MIC of mecillinam were both retained in the final model, and thus associated with treatment failure. An ESBL-producing strain was associated with a three-fold risk, and each doubling of mecillinam MIC (from ≤1 mg/L), was associated with a two-fold risk of treatment failure. Thus, the treatment failure rate for ESBL-positive strains was substantially greater than for ESBL-negative strains expressing the same mecillinam MIC (Figure 1).

Non-mecillinam treatment group. Inappropriate initial treatment was the only variable retained in the final model and was strongly associated with treatment failure. If this variable was omitted from analysis, the final model would include ESBL status (OR = 2.4, CI 1.03–5.5, p = 0.04), trimethoprim resistance

Table 1. Prevalence of resistance in ESBL-producing and non-ESBL-producing *E. coli*.

Resistance to	ESBL-producing *E. coli* (n = 81)	Non-ESBL-producing *E. coli* (n = 262)	p-value
Ampicillin	100%	40%	<0.001
Mecillinam	6.2%	0.4%	0.001
Trimethoprim	74%	29%	<0.001
Trimethoprim-sulfamethoxazole	72%	27%	<0.001
Nitrofurantoin	1.2%	0.0%	0.24
Ciprofloxacin	53%	7.7%	<0.001
Gentamicin	38%	5.2%	<0.001
Cefuroxime	98%	2.4%	<0.001
Cefotaxime	98%	0%	<0.001

doi:10.1371/journal.pone.0085889.t001

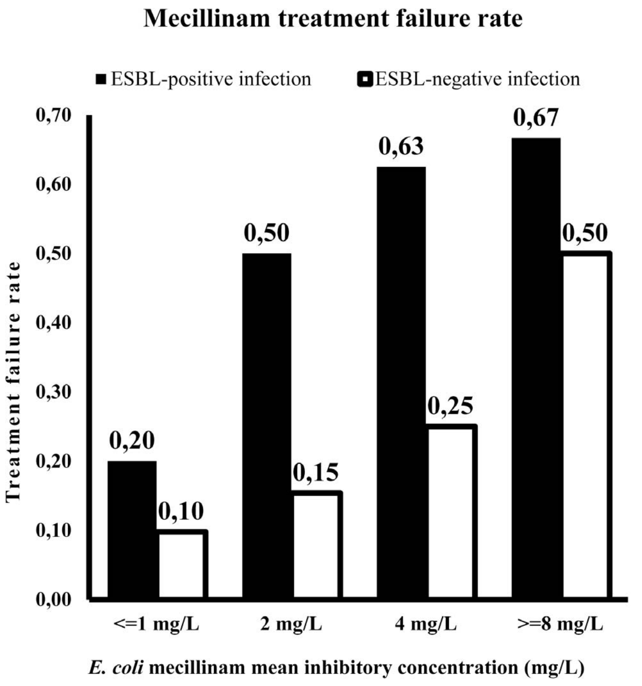

Figure 1. Mecillinam treatment failure rate among patients with community-acquired urinary tract infection caused by ESBL-producing and non-ESBL-producing *E. coli* with different mecillinam mean inhibitory concentrations.
doi:10.1371/journal.pone.0085889.g001

(OR = 6.4, CI 3.1–13.2, $p<0.001$) and treatment with nitrofurantoin (OR = 0.25, CI 0.08–0.8, $p = 0.02$).

The results of the multivariable analysis did not change significantly when i) the four patients with *in-vitro* mecillinam resistant microbes were excluded from the analysis in the mecillinam treatment group in the final model, ii) participants with a recent (≤1 month) history of UTI were excluded or iii) when the variables age, gender and number of UTIs during the past year were included in the final model.

Discussion

To our knowledge this is the first population-based study on the clinical efficacy of mecillinam in CA-UTI. We observed a significantly higher rate of mecillinam treatment failure in patients with a CA-UTI caused by ESBL-producing *E. coli* compared to non-ESBL-producing strains. The ESBL-producing strains were dominated by CTX-M type 1 and 9, in accordance with the current national and international situation [25,26].

There are several possible explanations for the high rate of mecillinam treatment failures in ESBL-producing *E. coli.* Firstly, we observed that the mean MIC of mecillinam in ESBL-producing strains was higher than in non-producers. A doubled MIC of mecillinam was associated with a two-fold risk of treatment failure in both univariate and multivariable analysis (Figure 1). This observation suggests that increasing the mecillinam dose or the dosing frequency might have reduced the treatment failure rate because the bactericidal activity of betalactam antibiotics is dependent on the time period the drug concentration exceeds the actual MIC at the infection site [27]. This notion is also supported by urine concentration measurements of mecillinam in healthy adults showing that a sensitive *E. coli* population should be suppressed by mecillinam in urine throughout a 24-h period if 400 mg pivmecillinam is given thrice daily [28]. Only 200 mg TID was prescribed to most study patients in accordance with Norwegian guidelines. Moreover, Monte Carlo simulations run to predict serum concentrations after 400 mg pivmecillinam given per os TID also support a higher dosage [29]. These simulations showed that this dose will only achieve a serum concentration above MIC for more than 40% of the time if MIC ≤0.25 mg/L. This is lower than for most ESBL-producing strains and supports the fact that that treatment failures can occur because of low dosing of mecillinam. Mecillinam and active metabolites accumulate in urine and a reduced antimicrobial potency of mecillinam would especially occur towards bacteria with slightly elevated MICs in upper urinary tract infections where

Table 2. Relevant patient characteristics in the mecillinam treatment group and univariate analysis of risk factors for treatment failure[a].

Characteristic	Treatment failure n = 34	Treatment success n = 124	Crude OR	95% CI	p-value
Age in years, mean ± SD	53±17	61±19	0.98	0.96–0.998	0.03
Female gender (%)	32 (94)	116 (94)	1.1	0.22–5.5	1.0
Number of urinary tract infections during past year, mean ± SD[b]	1.0±1.4	1.2±1.5	0.91	0.69–1.2	0.51
Total prescribed dose of antimicrobial agent in DDD (median, IQR)[c]	6.7 (6.7–10)	6.7 (6.7–10)	0.82	0.66–1.0	0.13
ESBL-producing strain (%)	18 (53)	23 (19)	4.9	2.2–11	<0.001
Mecillinam MIC (mg/L) (median IQR)[d]	2 (≤1–4)	≤1 (≤1–≤1)	1.3	1.1–1.5	<0.001
Strain resistant to initial treatment (mecillinam) (%)	4 (12)	0 (0)	–	–	0.002
Strain resistant to ampicillin (%)	26 (76)	57 (46)	3.8	1.6–9.1	0.002
Strain resistant to ciprofloxacin (%)	12 (35)	19 (16[e])	3.0	1.3–7.0	0.01

[a]Data are presented as the absolute number of patients unless specifically noted.
[b]To quantify the number of UTIs for each patient in the preceding year, the number of prescriptions of three antimicrobial agents–trimethoprim, mecillinam, and nitrofurantoin–were counted. In Norway, these agents are first choices for UTI treatment and are not used for other infections.
[c]OR is per increase of one defined daily dose (DDD) (One DDD = 600 mg of pivmecillinam), IQR = inter-quartile range.
[d]MIC = minimal inhibitory concentration.
[e]Missing information on two patients.
doi:10.1371/journal.pone.0085889.t002

Table 3. Relevant patient characteristics in the non-mecillinam treatment group and univariate analysis of risk factors for treatment failure[a].

Characteristic	Treatment failure, n = 67	Treatment success n = 118	Crude OR	95% CI	p-value
Age in years, mean ± SD	57±18	61±16	0.99	0.97–1.0	0.14
Female gender (%)	54 (81)	98 (83)	0.85	0.39–1.8	0.68
Number of urinary tract infections during past year, mean ± SD[b]	1.0±1.8	1.1±1.7	0.96	0.80–1.1	0.34
Total dispensed dose of antimicrobial agent in DDD (median, IQR)[c]	5.6 (4.0–7.0)	5.6 (4.5–6.3)	0.95	0.86–1.1	0.33
ESBL-producing strain (%)	25 (37)	15 (13)	4.1	2.0–8.5	<0.001
Strain resistant to initial treatment (%)	45 (68[d])	8 (6.8)	29	12–71	<0.001
Strain resistant to ampicillin (%)	52 (78)	50 (42)	4.7	2.4–9.3	<0.001
Strain resistant to ciprofloxacin (%)	21 (31)	11 (9.3)	4.4	2.0–10	<0.001
Strain resistant to trimethoprim (%)	46 (69)	28 (24)	7.0	3.6–13	<0.001
Treatment					
- Treated with trimethoprim (%)	41 (61)	66 (56)	1.2	0.67–2.3	0.49
- Treated with a quinolone (%)	5 (7.5)	12 (10)	0.71	0.24–2.1	0.54
- Treated with nitrofurantoin (%)	5 (7.5)	21 (18)	0.37	0.13–1.0	0.052
- Treated with another antibiotic (including combinations)[e] (%)	16 (24)	19 (16)	1.6	0.78–3.4	0.19

[a]Data are presented as the absolute number of patients unless specifically noted.
[b]To quantify the number of UTIs for each patient in the preceding year, the number of prescriptions of three antimicrobial agents–trimethoprim, mecillinam, and nitrofurantoin–were counted. In Norway, these agents are first choices for UTI treatment and are not commonly used for other infections.
[c]OR is per increase of one defined daily dose (DDD). IQR = inter-quartile range.
[d]Missing information on one patient.
[e]The other group consist of patients treated with (numbers of patients in parentheses): trimethoprim-sulfamethoxazole (16), intravenous treatment (9), amoxicillin (5), cefalexin (3), pivmecillinam and nitrofurantoin (1) and pivmecillinam and trimethoprim (1).
doi:10.1371/journal.pone.0085889.t003

accumulation of mecillinam in the urine is less pronounced [28,30,31].

The other variable found to be associated with treatment failure in multivariable analysis was ESBL-status itself (Figure 1). This is consistent with previous in vitro studies on the activity of mecillinam against ESBL-producing *E. coli* showing that mecillinam is not stable against ESBLs [2,13,14]. ESBL-producing strains have an inoculum dependent MIC for mecillinam. Agar dilution analyses of CTX-M producing *E. coli* with and without clavulanic acid added showed a significant inoculum effect on the MIC of mecillinam that was reversed by clavulanate [13,14]. An inoculum of 10^6 CFU/spot gave an approximately 100-fold increase in mecillinam MIC compared to the standard inoculum (10^4 CFU/spot). Interestingly, recently published time-kill analyses showed a significant bactericidal activity in only 7/48 (15%) of CTX-M producing *E. coli* strains even with the addition of clavulanic acid [13]. In vitro antimicrobial susceptibility tests are mostly based on bacteriostatic rather than bactericidal activity and the observed reduced bactericidal effect of mecillinam against ESBL-producing *E. coli* may therefore pass unrecognised.

Our results may seem to contradict the recently published 100% mecillinam treatment success rate in seven patients with ESBL-producing *E. coli* [10]. However, six of those strains had a MIC of mecillinam ≤1 mg/L while the last one had a MIC of 2 mg/L. Among our fifteen ESBL-producing strains with a mecillinam MIC ≤1 mg/L, treatment failure was only noted in three (20%). Thus, our results are compatible with the observations made in this small case study [10].

Other studies investigating effect of mecillinam in the treatment of (non-ESBL) UTI have reported lower overall treatment failure rates than ours [32,33], while others have reported comparable results [34,35]. Several factors may have contributed to an overall

Table 4. Independent risk factors of treatment failure in the mecillinam and the non-mecillinam treatment group.

Treatment group and variable	Level	Adjusted OR	95% CI	p-value
**Mecillinam group**				
- ESBL-producing strain	Yes/no	3.2	1.3–7.8	0.009
- Mecillinam MIC[a]	Doubling of MIC	2.0	1.4–3.0	<0.001
**Non-mecillinam group**				
- Strain resistant to initial treatment	Yes/no	29.5	12–71	<0.001

[a]For each doubling concentration starting at 1 mg/L which is the lowest level reported by the VITEK-2 (BioMerieux) system.
doi:10.1371/journal.pone.0085889.t004

high rate of treatment failure in the present study. Firstly, the inclusion criteria did not exclude complicated UTIs. Secondly, only bacteriologically verified UTIs were included. This criterion selects towards complicated UTI as the diagnosis of sporadic CA-UTI in women is often not supported by urine culture in Norway. Thirdly, a large proportion of the infections were caused by ESBL-producing strains with multiple drug resistance. Fourthly, patients with new UTIs occurring within two weeks from the index UTI and receiving a new prescription would have been classified as treatment failures under this study protocol. Given that 72% of treatment failures occurred within seven days this effect is probably small. Finally, the mean age of the study population was relatively high compared to other studies probably due to indications for culturing as mentioned above [36].

Our study was observational and associations between variables and treatment failure are therefore susceptible to bias. Only 37% of invited patients accepted the invitation to participate in the study and 40% of these patients did not receive empirical treatment. We have limited information about non-participants except for age, but assume this is a non-differential bias since both treatment groups probably are affected the same way. Another bias that may affect patients in different treatment groups differently is side effects resulting in new prescriptions that will be recorded as treatment failures. Furthermore, some patients may have been contacted by their doctor's practice staff when susceptibility testing identified bacterial resistance against the initial antimicrobial agent. This may have resulted in additional prescription indicating initial treatment failure even if the patient had clinical improvement. However, the significant association between clinical outcome recorded during interviews and data from the prescription database strongly indicates that these effects have been limited and that change of treatment in most cases was guided by patient symptoms. This underlines the reliability of a repeated prescription within 14 days as a valid surrogate marker for treatment failure. The patients were not randomized between treatment schemes. However, it is unlikely that this has affected the overall outcome since ESBL status was not known prior to treatment and patients with prior ESBL-positive infection were not included. Furthermore, the choice of treatment (type and duration) did not seem to be affected by ESBL status (data not shown). Finally, TEM-1 has a hydrolytic activity against mecillinam [14]. This enzyme may be present in ampicillin resistant strains including ESBL-producing strains. The OR for mecillinam treatment failure in non-ESBL producing ampicillin resistant versus non-ampicillin resistant strains was 2.0 (95% CI: 0.68–5.7, $p = 0.21$). Characterization of mechanisms of ampicillin resistance or identification of possible narrow spectrum bla_{TEM} or bla_{SHV} genes in ESBL-producing strains was not performed and could not be accounted for in the analyses performed. Thus this is a potential source of bias in the study.

Mecillinam has been proposed as an anti-ESBL agent [12]. The present study indicates that mecillinam with the current dosing (200 mg TID of pivmecillinam) has limited efficacy against infections caused by ESBL-producing *E. coli*. Although this is an observational study, we suggest that per oral mecillinam (i.e. pivmecillinam) should only be prescribed in uncomplicated UTIs caused by ESBL-producing *E. coli* if no other per oral options are available. We also suggest that higher doses of pivmecillinam than usually prescribed in Norway (200 mg TID) should be used because of the observed MIC-dependant efficacy. This is in particular relevant for patient at high risk of UTI caused by an ESBL-producing strains [16]. Significantly higher doses are manageable since pivmecillinam has a low toxicity. Our data also suggest that the mecillinam MIC break points for ESBL-producing *E. coli* should be reconsidered because of its reduced clinical efficacy and bactericidal effect against these strains.

Importantly the study results do not affect mecillinam's status as a first line drug in the empirical treatment of CA-UTI. The overall treatment failure rate was lower in patients receiving mecillinam (22%) than for patients in the non-mecillinam treatment (36%). This difference between the mecillinam and non-mecillinam group was valid also with different ESBL status (44% vs. 63% treatment failure in the ESBL group and 14% vs. 29% treatment failure in the non-ESBL group for patients in the mecillinam group and non-mecillinam group, respectively). This is probably because of the high prevalence of resistance to the other first-line per oral antibacterial drugs most commonly used against CA-UTI (Table 1).

In conclusion, we observed a high rate of mecillinam treatment failure in CA-UTI caused by ESBL-producing *E. coli* even for *in vitro* sensitive strains. The treatment failure of mecillinam was associated with ESBL-production per se as well as the increased MIC for mecillinam in ESBL-producers. Mecillinam is ecologically favourable and has a well documented effect in CA-UTI caused by non-ESBL producing *E. coli*. Further studies addressing the use of pivmecillinam against ESBL-producing *E. coli* with emphasis on optimal dosing and effect of combination therapy with β-lactamase inhibitors seem warranted.

Acknowledgments

We thank Anne Fritzvold, Bjørg Haldorsen, and Carina Thilesen for their excellent technical assistance and for the services of the Norwegian Prescription Database.

Author Contributions

Conceived and designed the experiments: ASO ASU PJ. Performed the experiments: ASO ASU PJ. Analyzed the data: ASO ASU SJ KL PAJ. Contributed reagents/materials/analysis tools: ASO ASU PJ. Wrote the paper: ASO ASU SJ KL PAJ.

References

1. Garau J (2008) Other antimicrobials of interest in the era of extended-spectrum beta-lactamases: fosfomycin, nitrofurantoin and tigecycline. Clin Microbiol Infect 14 Suppl 1: 198–202.
2. Sougakoff W, Jarlier V (2000) Comparative potency of mecillinam and other beta-lactam antibiotics against Escherichia coli strains producing different beta-lactamases. J Antimicrob Chemother 46 Suppl 1: 9–14.
3. Gupta K, Hooton TM, Naber KG, Wullt B, Colgan R et al. (2011) International clinical practice guidelines for the treatment of acute uncomplicated cystitis and pyelonephritis in women: A 2010 update by the Infectious Diseases Society of America and the European Society for Microbiology and Infectious Diseases. Clin Infect Dis 52: e103–e120.
4. Dewar S, Reed LC, Koerner RJ (2013) Emerging clinical role of pivmecillinam in the treatment of urinary tract infection in the context of multidrug-resistant bacteria. J Antimicrob Chemother.
5. Sullivan A, Edlund C, Svenungsson B, Emtestam L, Nord CE (2001) Effect of perorally administered pivmecillinam on the normal oropharyngeal, intestinal and skin microflora. J Chemother 13: 299–308.
6. Sullivan A, Fianu-Jonasson A, Landgren BM, Nord CE (2005) Ecological effects of perorally administered pivmecillinam on the normal vaginal microflora. Antimicrob Agents Chemother 49: 170–175.
7. Kahlmeter G, Poulsen HO (2012) Antimicrobial susceptibility of Escherichia coli from community-acquired urinary tract infections in Europe: the ECO.SENS study revisited. Int J Antimicrob Agents 39: 45–51.
8. NORM/NORM-VET 2010 (2011) Usage of Antimicrobial Agents and Occurrence of Antimicrobial Resistance in Norway. Tromsø/Oslo 2011. ISSN:1502–2307 (print)/1890–9965 (electronic).
9. Tarnberg M, Ostholm-Balkhed A, Monstein HJ, Hallgren A, Hanberger H et al. (2011) In vitro activity of beta-lactam antibiotics against CTX-M-producing Escherichia coli. Eur J Clin Microbiol Infect Dis 30: 981–987.

10. Titelman E, Iversen A, Kalin M, Giske CG (2012) Efficacy of pivmecillinam for treatment of lower urinary tract infection caused by extended-spectrum beta-lactamase-producing Escherichia coli and Klebsiella pneumoniae. Microb Drug Resist 18: 189–192.
11. Auer S, Wojna A, Hell M (2010) Oral treatment options for ambulatory patients with urinary tract infections caused by extended-spectrum-beta-lactamase-producing Escherichia coli. Antimicrob Agents Chemother 54: 4006–4008.
12. Wootton M, Walsh TR, Macfarlane L, Howe RA (2010) Activity of mecillinam against Escherichia coli resistant to third-generation cephalosporins. J Antimicrob Chemother 65: 79–81.
13. Lampri N, Galani I, Poulakou G, Katsarolis I, Petrikkos G et al. (2012) Mecillinam/clavulanate combination: a possible option for the treatment of community-acquired uncomplicated urinary tract infections caused by extended-spectrum beta-lactamase-producing Escherichia coli. J Antimicrob Chemother 67: 2424–2428.
14. Thomas K, Weinbren MJ, Warner M, Woodford N, Livermore D (2006) Activity of mecillinam against ESBL producers in vitro. J Antimicrob Chemother 57: 367–368.
15. Nicolle LE, Mulvey MR (2007) Successful treatment of ctx-m ESBL producing Escherichia coli relapsing pyelonephritis with long term pivmecillinam. Scand J Infect Dis 39: 748–749.
16. Soraas A, Sundsfjord A, Sandven I, Brunborg C, Jenum PA (2013) Risk Factors for Community-Acquired Urinary Tract Infections Caused by ESBL-Producing Enterobacteriaceae -A Case-Control Study in a Low Prevalence Country. PLoS One 8: e69581.
17. Berg C, Furu K, Mahic M, Litleskare I, Rønning M et al. (2011) The Norwegian Prescription Database 2006–2010: The Norwegian Institute of Public Health. ISBN: 978-82-8082-458-5.
18. European Committee on Antimicrobial Susceptibility Testing. Breakpoint tables for interpretation of MICs and zone diameters. Available: http://www.eucast.org/Accessed 1 October 2013.
19. Birkett CI, Ludlam HA, Woodford N, Brown DF, Brown NM et al. (2007) Real-time TaqMan PCR for rapid detection and typing of genes encoding CTX-M extended-spectrum beta-lactamases. J Med Microbiol 56: 52–55.
20. Tofteland S, Haldorsen B, Dahl KH, Simonsen GS, Steinbakk M et al. (2007) Effects of phenotype and genotype on methods for detection of extended-spectrum-beta-lactamase-producing clinical isolates of Escherichia coli and Klebsiella pneumoniae in Norway. J Clin Microbiol 45: 199–205.
21. Lawrenson RA, Logie JW (2001) Antibiotic failure in the treatment of urinary tract infections in young women. J Antimicrob Chemother 48: 895–901.
22. Goettsch WG, Janknegt R, Herings RM (2004) Increased treatment failure after 3-days' courses of nitrofurantoin and trimethoprim for urinary tract infections in women: a population-based retrospective cohort study using the PHARMO database. Br J Clin Pharmacol 58: 184–189.
23. Bjerrum L, Dessau RB, Hallas J (2002) Treatment failures after antibiotic therapy of uncomplicated urinary tract infections. A prescription database study. Scand J Prim Health Care 20: 97–101.
24. Viera AJ, Garrett JM (2005) Understanding interobserver agreement: the kappa statistic. Fam Med 37: 360–363.
25. Livermore DM (2012) Current epidemiology and growing resistance of gram-negative pathogens. Korean J Intern Med 27: 128–142.
26. Naseer U, Sundsfjord A (2011) The CTX-M conundrum: dissemination of plasmids and Escherichia coli clones. Microb Drug Resist 17: 83–97.
27. Craig WA (2003) Basic pharmacodynamics of antibacterials with clinical applications to the use of beta-lactams, glycopeptides, and linezolid. Infect Dis Clin North Am 17: 479–501.
28. Kerrn MB, Frimodt-Moller N, Espersen F (2004) Urinary concentrations and urine ex-vivo effect of mecillinam and sulphamethizole. Clin Microbiol Infect 10: 54–61.
29. Jensen KS, Henriksen AS, Frimodt-Moeller N (2008) Pivmecillinam: estimation of adequate dosage for susceptible and ESBL-producing *E. coli* by Monte Carlo PK/PD simulation. Abstracts of the 18nd European Congress of Clinical Microbiology and Infectious Diseases, Barcelona, Spain, 2008 Abstract P1255 European Society of Clinical Microbiology and Infectious Diseases, Basel, Switzerland.
30. Hvidberg H, Struve C, Krogfelt KA, Christensen N, Rasmussen SN et al. (2000) Development of a long-term ascending urinary tract infection mouse model for antibiotic treatment studies. Antimicrob Agents Chemother 44: 156–163.
31. Frimodt-Moller N (2002) Correlation between pharmacokinetic/pharmacodynamic parameters and efficacy for antibiotics in the treatment of urinary tract infection. Int J Antimicrob Agents 19: 546–553.
32. Bjerrum L, Gahrn-Hansen B, Grinsted P (2009) Pivmecillinam versus sulfamethizole for short-term treatment of uncomplicated acute cystitis in general practice: a randomized controlled trial. Scand J Prim Health Care 27: 6–11.
33. Menday AP (2000) Comparison of pivmecillinam and cephalexin in acute uncomplicated urinary tract infection. Int J Antimicrob Agents 13: 183–187.
34. Ferry SA, Holm SE, Stenlund H, Lundholm R, Monsen TJ (2007) Clinical and bacteriological outcome of different doses and duration of pivmecillinam compared with placebo therapy of uncomplicated lower urinary tract infection in women: the LUTIW project. Scand J Prim Health Care 25: 49–57.
35. Nicolle LE, Madsen KS, Debeeck GO, Blochlinger E, Borrild N et al. (2002) Three days of pivmecillinam or norfloxacin for treatment of acute uncomplicated urinary infection in women. Scand J Infect Dis 34: 487–492.
36. Raz R, Gennesin Y, Wasser J, Stoler Z, Rosenfeld S et al. (2000) Recurrent urinary tract infections in postmenopausal women. Clin Infect Dis 30: 152–156.
