# Supplementary material for: Fecal carriage of extended spectrum β-lactamase producing Escherichia coli and Klebsiella pneumoniae after urinary tract infection – A three year prospective cohort study
Source: PLoS One. 2017 Mar 7;12(3):e0173510. doi: 10.1371/journal.pone.0173510 (PMC5340397; doi:10.1371/journal.pone.0173510)
Supplement: S4 Text — (PDF) [file pone.0173510.s009.pdf]

Risk Factors for Community-Acquired Urinary Tract Infections Caused by ESBL-Producing *Enterobacteriaceae* –A Case–Control Study in a Low Prevalence Country

Arne Søraas[1]*, Arnfinn Sundsfjord[2,3], Irene Sandven[4], Cathrine Brunborg[4], Pål A. Jenum[1]

1 Department of Medical Microbiology, Vestre Viken Hospital Trust, Bærum, Norway, 2 Department of Microbiology and Infection Control, Reference Centre for Detection of Antimicrobial Resistance, University Hospital of North Norway, Tromsø, Norway, 3 Department of Medical Biology, Research Group for Host-Microbe Interactions, Faculty of Health Sciences, University of Tromsø, Tromsø, Norway, 4 Unit of Biostatistics and Epidemiology, Oslo University Hospital, Oslo, Norway

Abstract

Community-acquired urinary tract infection (CA-UTI) is the most common infection caused by extended-spectrum β-lactamase (ESBL)-producing *Enterobacteriaceae,* but the clinical epidemiology of these infections in low prevalence countries is largely unknown. A population based case-control study was conducted to assess risk factors for CA-UTI caused by ESBL-producing *E. coli* or *K. pneumoniae*. The study was carried out in a source population in Eastern Norway, a country with a low prevalence of infections caused by ESBL-producing *Enterobacteriaceae*. The study population comprised 100 cases and 190 controls with CA-UTI caused by ESBL-producing and non-ESBL-producing *E. coli* or *K. pneumoniae,* respectively. The following independent risk factors of ESBL-positive UTIs were identified: Travel to Asia, The Middle East or Africa either during the past six weeks (Odds ratio (OR) = 21; 95% confidence interval (CI): 4.5–97) or during the past 6 weeks to 24 months (OR = 2.3; 95% CI: 1.1–4.4), recent use of fluoroquinolones (OR = 16; 95% CI: 3.2–80) and β-lactams (except mecillinam) (OR = 5.0; 95% CI: 2.1–12), diabetes mellitus (OR = 3.2; 95% CI: 1.0–11) and recreational freshwater swimming the past year (OR = 2.1; 95% CI: 1.0–4.0). Factors associated with decreased risk were increasing number of fish meals per week (OR = 0.68 per fish meal; 95% CI: 0.51–0.90) and age (OR = 0.89 per 5 year increase; 95% CI: 0.82–0.97). In conclusion, we have identified risk factors that elucidate mechanisms and routes for dissemination of ESBL-producing *Enterobacteriaceae* in a low prevalence country, which can be used to guide appropriate treatment of CA-UTI and targeted infection control measures.

Citation: Søraas A, Sundsfjord A, Sandven I, Brunborg C, Jenum PA (2013) Risk Factors for Community-Acquired Urinary Tract Infections Caused by ESBL-Producing *Enterobacteriaceae* –A Case–Control Study in a Low Prevalence Country. PLoS ONE 8(7): e69581. doi:10.1371/journal.pone.0069581

Editor: Jan Kluytmans, Amphia Ziekenhuis, Netherlands

Received May 14, 2013; **Accepted** June 14, 2013; **Published** July 23, 2013

Copyright: © 2013 Søraas et al. This is an open-access article distributed under the terms of the Creative Commons Attribution License, which permits unrestricted use, distribution, and reproduction in any medium, provided the original author and source are credited.

Funding: The authors have no support or funding to report.

Competing Interests: The authors have declared that no competing interests exist.

* E-mail: arne@meg.no

Introduction

During the past 15 years, we have observed a worldwide dissemination of infections caused by CTX-M extended-spectrum β-lactamase (ESBL)-producing *Enterobacteriaceae* [1]. These infections are associated with increased mortality, morbidity, health care costs, and the need for broad-spectrum antibiotics [2]. Community-acquired urinary tract infection (CA-UTI) is the most common infection caused by ESBL-producing bacteria, but we have limited knowledge regarding the clinical epidemiology of these infections [1,3]. Most studies have focused on health care related infections and associated risk factors. Moreover, these studies have largely been based on information from medical records. Thus, information on possible risk factors not regularly noted in those records is sparse [4–20]. A large multinational survey of infections caused by ESBL-producing *Enterobacteriaceae* identified age ≥65 years, male sex and recent use of cephalosporins as independent risk factors for CA-ESBL infections [3]. However, the authors expressed a poor predictive value of their chosen model.

The present study was conducted in Norway. The yearly Norwegian nationwide antimicrobial resistance surveillance programme has shown a very low prevalence of infections caused by ESBL-producing *Enterobacteriaceae* [21]. A prevalence of 1.6% ESBL positive UTI in the Norwegian population was estimated for 2011. The prevalence is slowly increasing. A country with low prevalence of infections with ESBL-producing bacteria is well suited to identify risk factors for acquisition of ESBL, and a nationwide prescription database makes Norway suitable for the study of antibiotic use in detail [22]. Based on these advantages and patient interviews we aimed to investigate whether patients with ESBL positive CA-UTI have a different frequency of risk factors of CA-UTI as compared to patients with ESBL negative CA-UTI.

Materials and Methods

Design and Study Population

A case-control study was conducted at the Department of Medical Microbiology, Vestre Viken Hospital Trust situated in a mixed urban, suburban and rural area in the South-Eastern part of Norway. Our two laboratories analyse samples from in- and outpatients in an area comprising four hospitals and approximately 450.000 inhabitants (source population). The inclusion period was from February 2009 to April 2011.

The eligible population constituted all patients ≥18 years old with a urine culture yielding *E. coli* or *K. pneumoniae* >10,000 CFU/ml. The following exclusion criteria were used: i) patients who had lived in Norway for <1 year, ii) were unable to answer our questionnaire, iii) had previously diagnosed infection caused by ESBL-producing bacteria, and iv) patients with health care associated UTI (i.e., hospitalized or residing in a nursing home for >24 hours during the last 31 days).

The study population consisted of all patients willing to participate with ESBL-positive UTI (case group) and randomly selected patients with ESBL-negative UTI (control group) (Figure 1).

The patients received written information and were invited to participate by ordinary mail. Non-responders were contacted twice. Acceptance was given by returning a signed consent form.

Ethics statement. The study was approved by the Regional Committee for Medical and Health Research Ethics in South-Eastern Norway (reference number: 2009/2037 BS-08901b).

Data Collection

Urine cultivation and bacterial identification were performed using ChromID CPS3 agar and the VITEK-2 system (both BioMerieux, Marcy l'Etoile, France). Antimicrobial susceptibility testing and interpretations including ESBL screening were performed using VITEK-2 or agar disc diffusion method according to EUCAST recommendations and clinical breakpoints [23].

Isolates resistant to cefpodoxime, cefotaxime or ceftazidime were selected for confirmatory ESBL testing using the E-test system (AB-Biodisk, BioMerieux). ESBL genotype analysis was performed using PCR for $bla_{\mathrm{CTX\text{-}M}}$ detection and group assignment, as described [24]. Isolates negative for $bla_{\mathrm{CTX\text{-}M}}$ were analyzed using conventional bla_{TEM} and bla_{SHV} PCR and sequencing, as described [25].

A structured interview was performed by a trained investigator by telephone or in-person for community-based and hospitalized patients, respectively. The questionnaire was sent to the participants in advance and included questions regarding the infection for which they were included in the study, health condition (Charlson Comorbidity Index [26]), contact with the health care system in Norway and abroad (time and duration during the past 5 years), UTIs, antibiotic use, compliance with antibiotic prescriptions, prostate disease, use of a urinary catheter during the past year, oral and digestive health problems, international travel or residency lasting ≥24 hours during the past five years (time since returning home, duration and country), profession, personal hygiene, household members, pets, eating habits (meals per week of different foods and meals outside home), and recreational swimming during the past year (location, number of times and submergence of head).

In Norway antibiotics are available on prescription only. Date, type and amount of antibiotic dispensed during the past five years were obtained from The Norwegian Prescription Database [22]. Information about antibiotic use during hospitalization was obtained from medical records.

Information on previous infections with ESBL-producing bacteria was obtained from our laboratorys computer system.

Statistical Analysis

This case-control study was analysed using a pragmatic strategy, which means that priority was not given to a specific hypothesis.

Univariate analyses were performed using Student's t test, Pearson's chi-square test or Fisher's exact test when appropriate. The association between potential risk factors and infection caused by ESBL-producing *E. coli* or *K. pneumoniae* was quantified by odds ratio (OR) with 95% confidence interval (CI). Any variable with a $p<0.15$ from the univariate analysis was considered a candidate for the multivariate model. A manual backward stepwise

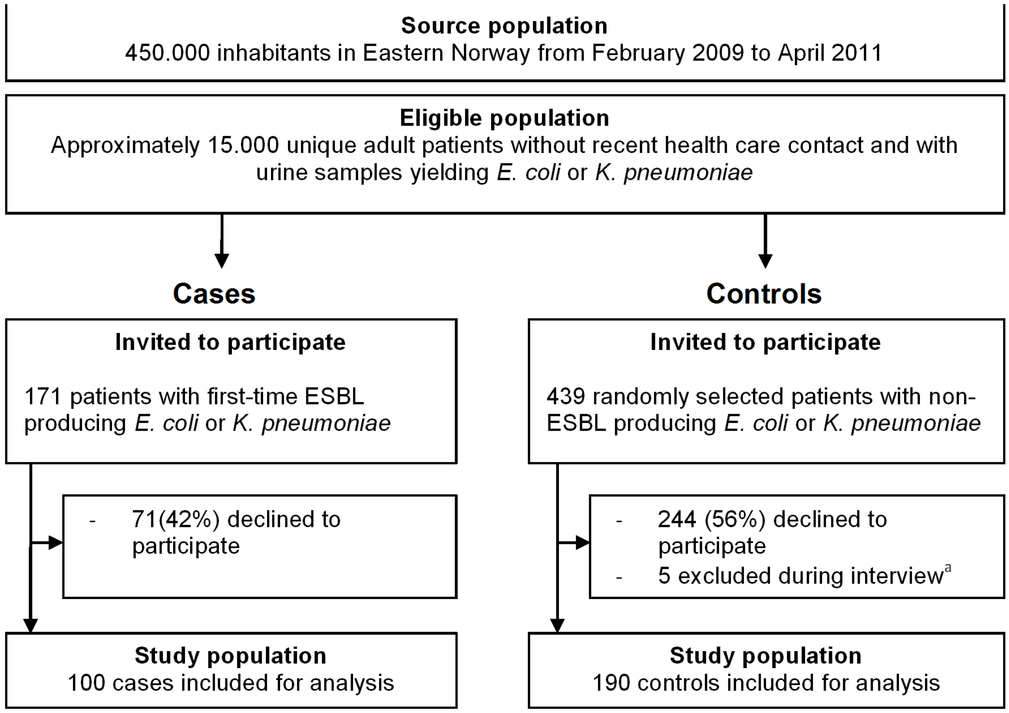

Figure 1. Selection of study population. [a]Dementia (n = 1), unable to reach by phone (n = 2) and death (n = 2).
doi:10.1371/journal.pone.0069581.g001

elimination procedure using a multivariate logistic regression model was performed to identify independent risk factors. Multivariate analyses were preceded by estimation of correlation between risk factors. Evaluation of the predictive accuracy of the models was assessed by calibration and discrimination. Calibration was evaluated by the Hosmer and Lemeshow goodness-of-fit test. A statistically non-significant Hosmer and Lemeshow result ($p>0.05$) suggests that the model predicts accurately on average. Discrimination was evaluated by analysis of the area under the ROC curve. We defined acceptable discriminatory capability as an area under the ROC curve greater than 0.7 [27]. Two-tailed *p* values of <0.05 were considered statistically significant. All statistical analyses were conducted using PASW statistics software, version 19.0 (IBM SPSS, Chicago, IL).

Results

Approximately 28,000 urine samples from 15,000 unique patients were submitted to our department during the inclusion period. A total of 359 (1.3%) samples yielded ESBL positive *E. coli* (n = 342) or *K. pneumoniae* (n = 17). After exclusion 171 subjects with ESBL UTI were invited to participate (case group). Also, 439 randomly selected control patients were invited to participate (Figure 1).

Relevant background characteristics of the participants are presented in Table 1. The cases and controls were in large similar. Significantly younger age and the presence of diabetes mellitus among cases were the two exceptions.

ESBL Genotyping

PCR and sequence analyses showed that 65%, 30%, and 5% of the ESBL isolates belonged to the CTX-M group 1, CTX-M group 9 and SHV group 5/12, respectively. TEM-type ESBLs were not detected.

Antibiotic Use and Antibiotic Resistance

Data on antibiotic use are presented in Table 2. More than 90% of the participants reported that they had completed all prescribed courses of antibiotics received during the past 5 years. Antibiotic use was more prevalent in the study population (59% during the past three months before the infection) than in the age-adjusted general Norwegian population (29% during the past year) – (data from the Norwegian Prescription Registry [22]). This difference was mainly due to increased use of antimicrobials used to treat UTIs in the study population.

In general, ESBL-producing isolates expressed more co-resistances compared to non-ESBL strains. For cases and controls the proportion of non-susceptible strains were 59% and 13% for ciprofloxacin, 78% and 24% for trimethoprim, 35% and 4% for gentamicin, 4% and 2% for nitrofurantoin and 4% and 3% for mecillinam, respectively.

Table 1. Demographic and clinical characteristics of the study population with and without ESBL positive urinary tract infection.[a]

Variable[b]	ESBL positive (n = 100)	ESBL negative (n = 190)	Crude OR	95% CI	p
Age in years, mean ± SD	55±19	64±17			<0.001
Female gender	88 (88%)	168 (88%)	0.96	0.45–2.0	0.92
Number of household members, mean ± SD	2.4±1.3	2.1±1.1			0.09
Pets in household	30 (30%)	44 (23%)	1.4	0.82–2.5	0.20
Infection caused by *Klebsiella pneumoniae*	5 (5%)	13 (7%)	0.72	0.25–2.1	0.54
Hospitalization past year[c]	21 (21%)	34 (18%)	1.2	0.66–2.2	0.52
Recurrent UTI[d]	17 (17%)	47 (25%)	0.62	0.34–1.2	0.13
Charlson index score ≥3	11 (11%)	25 (13%)	0.83	0.39–1.8	0.64
Pulmonary disease	13 (13%)	25 (13%)	0.99	0.48–2.0	0.98
Rheumatic disease	9 (9%)	33 (17%)	0.47	0.21–1.0	0.05
Malignancy	6 (6%)	9 (5%)	1.3	0.45–3.7	0.64
Diabetes mellitus	12 (12%)	9 (5%)	2.7	1.1–6.8	0.02
Gastrointestinal disease	14 (14%)	29 (15%)	0.90	0.45–1.8	0.76
Cardiac disease	13 (13%)	31 (17%)	0.76	0.38–1.5	0.44
Renal dysfunction	7 (7%)	10 (5%)	1.35	0.50–3.7	0.56
Hepatic dysfunction	1 (1%)	1 (1%)	1.90	0.12–31	1.00
Cerebrovascular disease	2 (2%)	10 (5%)	0.36	0.08–1.7	0.23
Urinary catheter at any time during past year	15 (15%)	25 (14%)	1.1	0.56–2.2	0.74

[a]Data are presented as the absolute number of patients with percentages in parentheses with the exception of age and household members, which is listed as mean value ± standard deviation (SD).
[b]Some variables have missing values (number of missing patients in parentheses): Household members (2), Charlson comorbidity index score (8) Pulmonary disease (2) Rheumatic disease (1), Malignancy (2), Diseases of the gastrointestinal tract (1), Cardiac disease (4), Renal dysfunction (1), Hepatic dysfunction (1), Cerebrovascular disease (2), Urinary catheter (6).
[c]Excluding the time period from 24 hours to 31 days before the urinary sample was taken. No patient had resided in a nursing home without being hospitalized in the time period.
[d]To quantify the number of UTIs for each patient in the preceding year, the number of prescriptions of three antimicrobial agents–trimethoprim, mecillinam, and nitrofurantoin–were counted. In Norway, these agents are first choices for UTI treatment and are not used for other infections. Recurrent UTI was defined as ≥3 UTIs during the past year.
doi:10.1371/journal.pone.0069581.t001

Table 2. Comparison of the antibiotic usage during the last 90 days prior to inclusion in the study population with and without ESBL positive urinary tract infection.

Antimicrobial agents[a]	ESBL positive (n = 100)	ESBL negative (n = 190)	Crude OR	95% CI	p
No antibiotic past 90 days[b]	38 (38%)	80 (42%)	0.84	0.51–1.4	0.50
Mecillinam	15 (15%)	45 (24%)	0.57	0.30–1.1	0.08
Macrolides	7 (7%)	5 (3%)	2.8	0.86–9.0	0.12
Tetracyclines	5 (5%)	6 (3%)	1.6	0.48–5.4	0.52
Fluoroquinolones	14 (14%)	3 (2%)	10	2.84–36	<0.001
Nitrofurantoin	8 (8%)	16 (8%)	0.95	0.39–2.3	0.90
Trimethoprim or trimethoprim/sulfamethoxazole	16 (16%)	42 (22%)	0.67	0.36–1.3	0.22
β-lactams except mecillinam[c]	18 (18%)	18 (9%)	2.1	1.0–4.2	0.04
- Phenoxymethylpenicillin	11 (11%)	12 (6%)	1.8	0.78–4.3	0.16
- Amoxicillin	3 (3%)	6 (3%)	0.95	0.23–3.9	1.0
- Cloxacillin	3 (3%)	1 (1%)	5.8	0.60–57	0.12
- Cephalexin	4 (4%)	2 (1%)	3.9	0.70–22	0.19
Methenamine hippurate	2 (2%)	15 (8%)	0.24	0.05–1.1	0.04

[a]Number of subjects who had used at least one dose in the past 90 days.
[b]Six cases and 17 controls received an antimicrobial agent at the day before the urinary sample only.
[c]Penicillin, amoxicillin, cloxacillin or cephalexin (some patients used more than one type).
doi:10.1371/journal.pone.0069581.t002

Risk Factor Analysis

The results of the univariate analyses on risk factors are presented in Table 3. Travelling to Asia, Middle East or Africa up to 2 years in the past, recreational swimming, eating dinner at restaurants and close occupational contact with humans were identified as significant risk factors for ESBL UTI. Interestingly, frequent consumption of fish meals (Figure 2), infrequent bath or shower and digestive problems seemed to have a protective effect.

The results of the multivariate analyses are presented in Table 4. Patients with an ESBL positive UTI had travelled 21 times more to Asia, Middle East or Africa during the past 6 weeks than patients with a non-ESBL UTI, and this was the strongest predictor for ESBL UTI. Travel to the same areas in the period from 6 weeks to 24 months in the past was to a lesser degree associated with ESBL UTI (OR 2.3, 95% CI: 1.2–4.4, p = 0.017). The variables regarding (time since) travel abroad were also analysed as continuous variables but this did not influence the results. Recreational freshwater swimming was identified as an independent risk factor, and patients with ESBL UTI had swum twice as frequent in freshwater as patients with ESBL negative UTI.

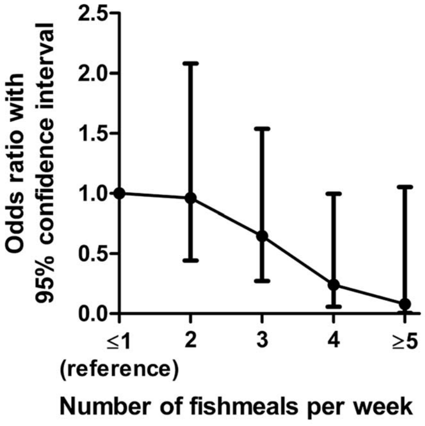

Figure 2. Decreasing risk[a] of ESBL-positive urinary tract infection with increasing number of fishmeals per week[b]. [a]Controlling for the variables: Travelling to Asia, Middle east or Africa, Use of fluoroquinolones the past 90 days, Use of β-lactams except mecillinam the past 90 days, Diabetes mellitus,Recreational freshwater swim past year and age. [b]Reference category: eating ≤1 fishmeal per week.
doi:10.1371/journal.pone.0069581.g002

Previously known risk factors such as recent antibiotic use and diabetes mellitus were also identified as independent risk factors. Age and weekly fish meals were found to be putative protective factors.

The final multivariate model was applied to participants with infection caused by *E. coli* only and this did not change any trends in the results (data not shown).

The Hosmer and Lemeshow goodness-of-fit test was not significant indicating a satisfactory fit of the model ($\chi^2 = 5.64$, df = 8, $p = 0.69$). The area under the ROC curve was 0.83 (95% CI: 0.79–0.88) indicating a good discriminative ability between ESBL-positive and ESBL-negative patients.

Discussion

This is to our knowledge the first population-based study to identify risk factors for acquisition of CA-ESBL infections in a low prevalence country. International travel was identified as the most important risk factor for ESBL positive CA-UTI in this study. Most travel-associated ESBL UTIs occurred during the first six weeks after returning home. This observation is consistent with previous studies and adds new information about the time course between colonization during travel and actual infection [5,28,29]. The area associated with the highest risk (Asia, Middle East and Africa) corresponds well with areas previously associated with a high rate of colonization in returning travellers [28].

This observation contrasts a recent French study. Nicolas-Chanoine and co-workers did not identify travelling abroad for

Table 3. Univariate comparison of risk factor exposition in the study population with and without ESBL-positive urinary tract infection.[a]

Variable[b]	ESBL positive (n = 100)	ESBL negative (n = 190)	Crude OR	95% CI	p
Travel destinations abroad within the past 6 weeks[c]					
- America or Oceania (including Japan)	0 (0%)	1 (1%)	0.65		1.00
- Asia, Middle East or Africa	23 (23%)	2 (1%)	28	6.5–122	<0.001
- Europe	11 (11%)	13 (7%)	1.7	0.72–3.9	0.22
Travel destinations abroad between the previous 6 weeks to 24 months[c]					
- America or Oceania (including Japan)	13 (13%)	17 (8.9%)	1.5	0.71–3.3	0.28
- Asia, Middle East or Africa	39 (39%)	36 (19%)	2.7	1.6–4.7	<0.001
- Europe	67 (67%)	108 (57%)	1.5	0.93–2.6	0.09
Travel destinations abroad between the previous 24 months to 5 years[c]					
- America or Oceania (including Japan)	10 (10%)	15 (7.9%)	1.3	0.56–3.0	0.54
- Asia, Middle East or Africa	26 (26%)	38 (20%)	1.4	0.79–2.5	0.24
- Europe	55 (55%)	92 (48%)	1.3	0.8–2.1	0.29
Recreational swimming past year					
- In seawater	68 (68%)	98 (52%)	2.0	1.2–3.3	0.01
- In freshwater	26 (26%)	30 (16%)	1.9	1.0–3.4	0.04
- In swimming pool	53 (53%)	78 (41%)	1.6	0.99–2.6	0.05
- Usually submerges head during recreational swimming	41 (41%)	56 (30%)	1.6	0.97–2.7	0.06
Eating habits					
- Number of fish meals per week, mean ±SD	2.1±1.1	2.7±1.4	0.67	0.54–0.83	<0.001
- Number of meat meals per week, mean ±SD	3.5±1.4	3.3±1.3	1.1	0.94–1.3	0.22
- Organic food ≥1/week	24 (24%)	40 (22%)	1.2	0.66–2.1	0.58
- Dinner at a restaurant ≥2/month	29 (29%)	28 (15%)	2.4	1.3–4.3	0.003
- Prefers meat well done	33 (34%)	74 (40%)	0.77	0.46–1.3	0.33
Close occupational contact with humans[d]	29 (29%)	31 (17%)	2.1	1.2–3.7	0.01
Bath or shower ≤2 times/week	12 (12%)	44 (23%)	0.46	0.23–0.92	0.03
Oral/dental health problems	13 (13%)	28 (15%)	0.85	0.42–1.7	0.65
Digestive problems (constipation or diarrhoea)	25 (26%)	75 (40%)	0.51	0.30–0.87	0.01

[a]Data are presented as the absolute number of patients with percentages in parentheses with the exception of fish and meat meals, which is listed as mean value ± SD.
[b]Some variables have missing values (number of missing patients in parentheses). Usually submerges head during recreational swimming (7), Organic food (7), Dinner in restaurant (2), Prefers meat well done (8), Close occupational contact with humans (6), Bath or shower (3), Digestive problems (6).
[c]Only trips lasting >24 hours outside the Nordic countries (Norway, Denmark, Finland, Sweden and Iceland) are included.
[d]Self-reported close occupational contact with humans.
doi:10.1371/journal.pone.0069581.t003

Table 4. Independent risk factors of ESBL positive community acquired urinary tract infection identified using multivariate logistic regression analysis.

Variable	Level	Adjusted OR	95% CI	P
Travelling to Asia, Middle East or Africa[a]				
- During the past 6 weeks	yes/no	21	4.5–97	<0.001
- Between the previous 6 weeks to 24 months	yes/no	2.3	1.2–4.4	0.017
Use of fluoroquinolones the past 90 days	yes/no	16	3.2–80	<0.001
Use of β-lactams except mecillinam in the past 90 days	yes/no	5.0	2.1–12	<0.001
Diabetes mellitus	yes/no	3.2	1.0–11	0.051
Recreational freshwater swim past year	yes/no	2.1	1.0–4.3	0.040
Age	5 year increase	0.89	0.82–0.97	0.014
Number of fish meals per week	1 meal increase	0.68	0.51–0.90	0.008

[a]Only trips lasting >24 hours are included.
doi:10.1371/journal.pone.0069581.t004

>14 days during the past 6 months as a risk factor for an ESBL-positive (*bla*$_{CTX-M-15}$) infection in hospitalized patients [6]. In our study, travelling abroad for >14 days was a strong predictor of ESBL UTI when using *bla*$_{CTX-M-1}$ -positive infections as an endpoint (data not shown). It is likely that the importance of travel as a risk factor will differ between the French hospitalized population and the Norwegian non-hospitalized population in our study. Also, the proportion of ESBL-producing clinical isolates of *Enterobacteriaceae* in France is higher than in Norway [30]. Therefore, travel abroad from France will not have the same relative impact on the colonization and infection rate as travelling abroad from Norway. This emphasizes the importance of investigating these risk factors in a low prevalence area.

Recent antibiotic use is a known risk factor for infections caused by ESBL-positive bacteria [3,7,8,11,31]. We found that recent use of fluoroquinolones was strongly associated with an ESBL-positive UTI, supporting the results from several other studies [7,8,31].

Interestingly, the use of mecillinam as opposed to other β-lactams, was not associated with ESBL-positive CA-UTI. This may be because the oral formulation of mecillinam, pivmecillinam, is a pro-drug with minor effects on the intestinal flora [32]. Moreover, mecillinam has a selective activity against Gram-negative bacteria and is more stable against ESBL hydrolysis compared to most penicillins [33].

Recreational swimming in freshwater was identified as an independent risk factor for ESBL UTI. ESBL-producing bacteria like *E. coli* have been detected in environmental water [34–36]. Furthermore, outbreaks of *E. coli O157:H7* have been linked to swimming in contaminated freshwater [37]. Swimming may therefore be a risk factor for intestinal colonization with *E. coli* with ESBL and any subsequent UTI may be caused by a such newly acquired ESBL-producing strain from the water [38]. This finding highlights a possible link between environmental pollution and antimicrobial resistance, but will have to be substantiated before any conclusions can be drawn [39].

Interestingly, eating fish was associated with a reduced risk of ESBL UTI (Figure 2). Each weekly fish meal reduced the risk of an ESBL positive infection with about 30%. It is clear that eating habits influence the microbial flora in the gut [40]. However, whether eating fish may affect the resistance pattern of the gut microbial flora and potentially lower the risk of ESBL UTI remains speculative and eating fish may be a marker of a more fundamental risk factor not measured.

Retail chicken meat has recently been implicated as a possible source of ESBL-colonization [41]. We did not specifically investigate this possible risk factor, but ESBL-producing bacteria have only very rarely been found in the Norwegian food chain [42].

In our study, patients infected with an ESBL-producing *E. coli* or *K. pneumoniae* were significantly younger than the control patients. In two studies with similar design but including hospitalized patients, no association between age and ESBL positive infection was found [8,43]. This suggests that the epidemiology of ESBL infections differs in Norway or among non-hospitalized patients.

Limitations

Limitations include the possibility of selection bias due to non-participation and a potential problem with differential misclassification of exposure because the interviewers were not masked to the status of the patient being a case or a control. To minimize the latter the questionnaires were sent to the participants in advance and interview training was given.

We did not use the Friedman criteria for health care acquired infections and thus patients with health care system contact during the past 2–3 months and patients catheterized the past month were included for analysis [44]. Excluding these patients (n = 30) did, however, not change any trends in the results (data not shown).

Finally, our study may overestimate the use of antibiotics as a risk factor since patients in the control group, with susceptible bacteria, may be less likely to have used antibiotics. This is because non-ESBL *E. coli* and *K. pneumoniae* are more susceptible to antibiotics than ESBL-producers, and recently treated patients with such susceptible strains are therefore less likely to show up in the control group [45].

In summary, we have addressed the knowledge gap concerning risk factors for CA-UTIs caused by ESBL-producing bacteria [3]. Previously suspected risk factors for ESBL UTI have been supported and possible new ones uncovered. Our study shows that the predictive antimicrobial resistance pattern in uropathogenic *E. coli* is heavily influenced by the country the patient has recently visited [28,46]. Thus, information on recent travel is important when treating patients with serious infections that may involve this organism. Physicians in low-prevalence countries should consider ESBL when treating UTI in patients who have visited countries in Africa, The Middle East or Asia during the past six weeks [28,46].

An association between recreational swimming and ESBL UTI was detected. Further investigation to examine the possible negative impact of environmental pollution with ESBL-producing *Enterobacteriaceae* seems warranted.

Finally, eating fish regularly was associated with a protective effect against ESBL UTI. If this is confirmed in other studies, an interesting link between diet and infection has been established.

Acknowledgments

We thank Anne Fritzvold, Bjørg Haldorsen and Carina Thilesen for their excellent technical assistance and the Norwegian Prescription Database for good service.

Author Contributions

Conceived and designed the experiments: A. Søraas A. Sundsfjord PAJ. Performed the experiments: A. Søraas. Analyzed the data: A. Søraas A. Sundsfjord IS CB PAJ. Wrote the paper: A. Søraas A. Sundsfjord IS CB PAJ.

References

1. Pitout JD, Laupland KB (2008) Extended-spectrum beta-lactamase-producing Enterobacteriaceae: an emerging public-health concern. Lancet Infect Dis 8: 159–166.
2. Rottier WC, Ammerlaan HS, Bonten MJ (2012) Effects of confounders and intermediates on the association of bacteraemia caused by extended-spectrum beta-lactamase-producing Enterobacteriaceae and patient outcome: a meta-analysis. J Antimicrob Chemother 67: 1311–1320.
3. Ben-Ami R, Rodriguez-Bano J, Arslan H, Pitout JD, Quentin C, et al. (2009) A multinational survey of risk factors for infection with extended-spectrum beta-lactamase-producing enterobacteriaceae in nonhospitalized patients. Clin Infect Dis 49: 682–690.
4. Doi Y, Park YS, Rivera JI, Adams-Haduch JM, Hingwe A, et al. (2013) Community-associated extended-spectrum beta-lactamase-producing Escherichia coli infection in the United States. Clin Infect Dis 56: 641–648.
5. Laupland KB, Church DL, Vidakovich J, Mucenski M, Pitout JD (2008) Community-onset extended-spectrum beta-lactamase (ESBL) producing Escherichia coli: importance of international travel. J Infect 57: 441–448.

6. Nicolas-Chanoine MH, Jarlier V, Robert J, Arlet G, Drieux L, et al. (2012) Patient's origin and lifestyle associated with CTX-M-producing Escherichia coli: a case-control-control study. PLoS One 7: e30498.
7. Colodner R, Rock W, Chazan B, Keller N, Guy N, et al. (2004) Risk factors for the development of extended-spectrum beta-lactamase-producing bacteria in nonhospitalized patients. Eur J Clin Microbiol Infect Dis 23: 163–167.
8. Rodriguez-Bano J, Picon E, Gijon P, Hernandez JR, Ruiz M, et al. (2010) Community-onset bacteremia due to extended-spectrum beta-lactamase-producing Escherichia coli: risk factors and prognosis. Clin Infect Dis 50: 40–48.
9. Cassier P, Lallechere S, Aho S, Astruc K, Neuwirth C, et al. (2011) Cephalosporin and fluoroquinolone combinations are highly associated with CTX-M beta-lactamase-producing Escherichia coli: a case-control study in a French teaching hospital. Clin Microbiol Infect 17: 1746–1751.
10. Siedelman L, Kline S, Duval S (2012) Risk factors for community- and health facility-acquired extended-spectrum beta-lactamase-producing bacterial infections in patients at the University of Minnesota Medical Center, Fairview. Am J Infect Control 40: 849–853.
11. Azap OK, Arslan H, Serefhanoglu K, Colakoglu S, Erdogan H, et al. (2010) Risk factors for extended-spectrum beta-lactamase positivity in uropathogenic Escherichia coli isolated from community-acquired urinary tract infections. Clin Microbiol Infect 16: 147–151.
12. Kang CI, Wi YM, Lee MY, Ko KS, Chung DR, et al. (2012) Epidemiology and risk factors of community onset infections caused by extended-spectrum beta-lactamase-producing Escherichia coli strains. J Clin Microbiol 50: 312–317.
13. Kim YK, Pai H, Lee HJ, Park SE, Choi EH, et al. (2002) Bloodstream infections by extended-spectrum beta-lactamase-producing Escherichia coli and Klebsiella pneumoniae in children: epidemiology and clinical outcome. Antimicrob Agents Chemother 46: 1481–1491.
14. Park SH, Choi SM, Lee DG, Kim J, Choi JH, et al. (2011) Emergence of extended-spectrum beta-lactamase-producing escherichia coli as a cause of community-onset bacteremia in South Korea: risk factors and clinical outcomes. Microb Drug Resist 17: 537–544.
15. Tian SF, Chen BY, Chu YZ, Wang S (2008) Prevalence of rectal carriage of extended-spectrum beta-lactamase-producing Escherichia coli among elderly people in community settings in China. Can J Microbiol 54: 781–785.
16. Topaloglu R, Er I, Dogan BG, Bilginer Y, Ozaltin F, et al. (2010) Risk factors in community-acquired urinary tract infections caused by ESBL-producing bacteria in children. Pediatr Nephrol 25: 919–925.
17. Muvunyi CM, Masaisa F, Bayingana C, Mutesa L, Musemakweri A, et al. (2011) Decreased susceptibility to commonly used antimicrobial agents in bacterial pathogens isolated from urinary tract infections in Rwanda: need for new antimicrobial guidelines. Am J Trop Med Hyg 84: 923–928.
18. Kim B, Kim J, Seo MR, Wie SH, Cho YK, et al. (2013) Clinical characteristics of community-acquired acute pyelonephritis caused by ESBL-producing pathogens in South Korea. Infection.
19. Johnson SW, Anderson DJ, May DB, Drew RH (2013) Utility of a clinical risk factor scoring model in predicting infection with extended-spectrum beta-lactamase-producing enterobacteriaceae on hospital admission. Infect Control Hosp Epidemiol 34: 385–392.
20. Briongos-Figuero LS, Gomez-Traveso T, Bachiller-Luque P, Dominguez-Gil GM, Gomez-Nieto A, et al. (2012) Epidemiology, risk factors and comorbidity for urinary tract infections caused by extended-spectrum beta-lactamase (ESBL)-producing enterobacteria. Int J Clin Pract 66: 891–896.
21. NORM/NORM-VET 2010 (2011) Usage of Antimicrobial Agents and Occurrence of Antimicrobial Resistance in Norway. Tromsø/Oslo 2011. ISSN:1502-2307 (print)/1890-9965 (electronic).
22. Berg C, Furu K, Mahic M, Litleskare I, Rønning M, et al. (2011) The Norwegian Prescription Database 2006–2010: The Norwegian Institute of Public Health. ISBN: 978-82-8082-458-5.
23. European Committee on Antimicrobial Susceptibility Testing. Breakpoint tables for interpretation of MICs and zone diameters. Available: http://www.eucast.org/Accessed 13-5-22013.
24. Birkett CI, Ludlam HA, Woodford N, Brown DF, Brown NM, et al. (2007) Real-time TaqMan PCR for rapid detection and typing of genes encoding CTX-M extended-spectrum beta-lactamases. J Med Microbiol 56: 52–55.
25. Tofteland S, Haldorsen B, Dahl KH, Simonsen GS, Steinbakk M, et al. (2007) Effects of phenotype and genotype on methods for detection of extended-spectrum-beta-lactamase-producing clinical isolates of Escherichia coli and Klebsiella pneumoniae in Norway. J Clin Microbiol 45: 199–205.
26. Chaudhry S, Jin L, Meltzer D (2005) Use of a self-report-generated Charlson Comorbidity Index for predicting mortality. Med Care 43: 607–615.
27. Kleinbaum DG, Klein M (2010) Assessing Discriminatory Performance of a Binary Logistic Model: ROC curves. In: Kleinbaum DG, Klein M. Logistic Regression. A Self-Learning Text. Third edition. In. 346–386.
28. Tangden T, Cars O, Melhus A, Lowdin E (2010) Foreign travel is a major risk factor for colonization with Escherichia coli producing CTX-M-type extended-spectrum beta-lactamases: a prospective study with Swedish volunteers. Antimicrob Agents Chemother 54: 3564–3568.
29. Freeman JT, McBride SJ, Heffernan H, Bathgate T, Pope C, et al. (2008) Community-onset genitourinary tract infection due to CTX-M-15-Producing Escherichia coli among travelers to the Indian subcontinent in New Zealand. Clin Infect Dis 47: 689–692.
30. European Centre for Disease Prevention and Control (2011) Antimicrobial resistance surveillance in Europe 2010. Annual Report of the European Antimicrobial Resistance Surveillance Network (EARS-Net). Stockholm: ECDC.
31. Rodriguez-Bano J, Navarro MD, Romero L, Martinez-Martinez L, Muniain MA, et al. (2004) Epidemiology and clinical features of infections caused by extended-spectrum beta-lactamase-producing Escherichia coli in nonhospitalized patients. J Clin Microbiol 42: 1089–1094.
32. Sullivan A, Edlund C, Svenungsson B, Emtestam L, Nord CE (2001) Effect of perorally administered pivmecillinam on the normal oropharyngeal, intestinal and skin microflora. J Chemother 13: 299–308.
33. Wootton M, Walsh TR, Macfarlane L, Howe RA (2010) Activity of mecillinam against Escherichia coli resistant to third-generation cephalosporins. J Antimicrob Chemother 65: 79–81.
34. Chen H, Shu W, Chang X, Chen JA, Guo Y, et al. (2010) The profile of antibiotics resistance and integrons of extended-spectrum beta-lactamase producing thermotolerant coliforms isolated from the Yangtze River basin in Chongqing. Environ Pollut 158: 2459–2464.
35. Tacao M, Correia A, Henriques I (2012) Resistance to broad-spectrum antibiotics in aquatic systems: anthropogenic activities modulate the dissemination of blaCTX-MlLike genes. Appl Environ Microbiol 78: 4134–4140.
36. Colomer-Lluch M, Jofre J, Muniesa M (2011) Antibiotic resistance genes in the bacteriophage DNA fraction of environmental samples. PLoS One 6: e17549.
37. Samadpour M, Stewart J, Steingart K, Addy C, Louderback J, et al. (2002) Laboratory investigation of an E. coli O157:H7 outbreak associated with swimming in Battle Ground Lake, Vancouver, Washington. J Environ Health 64: 16–20, 26, 25.
38. Moreno E, Andreu A, Perez T, Sabate M, Johnson JR, et al. (2006) Relationship between Escherichia coli strains causing urinary tract infection in women and the dominant faecal flora of the same hosts. Epidemiol Infect 134: 1015–1023.
39. Guenther S, Ewers C, Wieler LH (2011) Extended-Spectrum Beta-Lactamases Producing E. coli in Wildlife, yet Another Form of Environmental Pollution? Front Microbiol 2: 246.
40. Angelakis E, Armougom F, Million M, Raoult D (2012) The relationship between gut microbiota and weight gain in humans. Future Microbiol 7: 91–109.
41. Kluytmans JA, Overdevest IT, Willemsen I, Kluytmans-van den Bergh MF, van der Zwaluw K, et al. (2013) Extended-spectrum beta-lactamase-producing Escherichia coli from retail chicken meat and humans: comparison of strains, plasmids, resistance genes, and virulence factors. Clin Infect Dis 56: 478–487.
42. NORM/NORM-VET 2011 (2012) Usage of Antimicrobial Agents and Occurrence of Antimicrobial Resistance in Norway. Tromsø/Oslo 2012. ISSN:1502-2307 (print)/1890-9965 (electronic).
43. Apisarnthanarak A, Kiratisin P, Saifon P, Kitphati R, Dejsirilert S, et al. (2007) Clinical and molecular epidemiology of community-onset, extended-spectrum beta-lactamase-producing Escherichia coli infections in Thailand: a case-case-control study. Am J Infect Control 35: 606–612.
44. Friedman ND, Kaye KS, Stout JE, McGarry SA, Trivette SL, et al. (2002) Health care–associated bloodstream infections in adults: a reason to change the accepted definition of community-acquired infections. Ann Intern Med 137: 791–797.
45. Harris AD, Karchmer TB, Carmeli Y, Samore MH (2001) Methodological principles of case-control studies that analyzed risk factors for antibiotic resistance: a systematic review. Clin Infect Dis 32: 1055–1061.
46. Moor CT, Roberts SA, Simmons G, Briggs S, Morris AJ, et al. (2008) Extended-spectrum beta-lactamase (ESBL)-producing enterobacteria: factors associated with infection in the community setting, Auckland, New Zealand. J Hosp Infect 68: 355–362.
